# Combining antigenic data from public sources gives an early indication of the immune escape of emerging virus variants

**DOI:** 10.1038/s41598-025-19578-3

**Published:** 2025-10-24

**Authors:** Antonia Netzl, Sina Türeli, Eric B. LeGresley, Barbara Mühlemann, Samuel H. Wilks, Derek J. Smith

**Affiliations:** 1 Centre for Pathogen Evolution, Department of Zoology, University of Cambridge, Cambridge, United Kingdom; 2 Institute of Virology, Charité - Universitätsmedizin Berlin, Corporate Member of Freie Universität Berlin, Humboldt-Universität zu Berlin, and Berlin Institute of Health, Berlin, 10117 Germany; 3 German Centre for Infection Research (DZIF), Partner Site Charité, Berlin, 10117 Germany

**Keywords:** SARS-CoV-2, BA.1, Public data, Public health, Immune escape, Antigenic cartography, Viral infection, Computational biology and bioinformatics, Viral infection, RNA vaccines

## Abstract

The rapid spread of the Omicron BA.1 (B.1.1.529.1) SARS-CoV-2 (Severe Acute Respiratory Syndrome Coronavirus 2) variant in 2021 resulted in international efforts to quickly assess its escape from immunity generated by vaccines and previous infections. Numerous laboratories published BA.1 neutralization data as preprints and reports. We collated this data in real time and regularly presented updates of the aggregated results in US, European and WHO research and advisory settings. Here, we retrospectively analyzed the accuracy of these aggregations from 85 different sources published during a time period from 2021/12/08 up to 2022/08/14. We found that the mean titer fold change from wild type-like variants to BA.1, a standard measure of a variant’s immune escape, remained stable after the first 15 days of data reporting in people who were twice vaccinated, and incoming data increased the confidence in this quantity. Further, it is possible to build reliable, stable antigenic maps from this collated data already after one month of incoming data. We here demonstrate that combining early reports from variable, independent sources can rapidly indicate a new virus variant’s immune escape and can therefore be of immense benefit for public health.

## Introduction

The WHO classified the Omicron BA.1 variant (B.1.1.529+BA.1) as a Variant of Concern (VoC) on November 26, 2021^1^, and BA.1 quickly replaced Delta as the worldwide dominant variant. Since then, SARS-CoV-2 continues evolving and other variants took over. The rapid emergence and replacement of the dominant SARS-CoV-2 variant required quick reactions and global public health efforts to assess its immune escape in different contexts of vaccination and infection history. At the time of BA.1’s emergence, multiple laboratories rapidly produced virus neutralization data with diverse serum and variant panels and released them quickly as preprints or preliminary reports for public use, before journal publication. To aggregate the information from individual sources for research and policy guidance, we incrementally collected and summarized available data in openly accessible documents between December 2021 and August 2022^2,3^. This open and collaborative approach to science in response to the COVID-19 (Coronavirus disease-19) pandemic was crucial for informed public health policies. Here, we retrospectively analyze the data we collated in real time with a focus on how quickly summary results of BA.1’s immune escape reach a level of confidence relevant for public health guidance.

In late 2021, population immunity against SARS-CoV-2 in Europe and the US consisted of people infected with Wuhan-1/D614G, Alpha, Beta or Delta, and a large proportion of recipients of the two dose Wu-1 (Wuhan-1) vaccines. Wu-1 vaccines performed exceptionally well against the early SARS-CoV-2 variants, showing that up to that point the antigenic evolution of SARS-CoV-2 was moderate and did not necessitate a vaccine strain update^4^. BA.1 started to circulate around the time the recommendation for a third vaccine, or 1^st^ booster dose, was issued^5,6^. Its rapid takeover and infection of Wu-1 vaccinated people raised concerns about the protection conferred by an additional Wu-1 vaccine dose^7^.

Reacting quickly to the emergence of a virus variant is essential to keep infections, hospitalizations and deaths low. Such reactions include the assessment of the novel variant’s properties, including its transmissibility, severity, and potential to escape population immunity to inform public health decisions, such as advising for a vaccine strain update or implementing non-pharmaceutical interventions. Ideally, data to inform such decisions should be from controlled, reliable trials with large numbers of, preferably randomized, subjects. However, this type of data usually takes considerable time to generate which impedes a quick emergency response. With regards to the assessment of BA.1’s ability to escape population immunity, independent research and public health laboratories generated data quickly, albeit with smaller sample sizes and at times substantial variations across laboratories due to assay type, virus isolates, and type of and time since exposure of sera. After BA.1’s emergence, we responded to the quick action by the multiple individual laboratories and collated the not yet peer reviewed data that was published on preprint servers in real time. Here, we show that the limitation of small sample sizes and variation of data from independent sources can be overcome when combining data from multiple independent laboratories, giving a rapid indication of a variant’s escape potential to further public health decisions. Incremental versions of these data and analyses^2^ were frequently presented in national (UK and US), regional (WHO Europe) and global (WHO) scientific and public health fora as part of the scientific and public health response to BA.1^8–10^. Now, using publicly available data from 2021/12/08 up to 2022/08/14, we show that collated data from different sources can be used to produce stable vaccine escape measures soon after variant emergence, providing valuable information for a quick response to emergencies.

## Results

### Data collection

We analyzed Omicron BA.1 neutralization geometric mean titers (GMT) from 85 different sources which at the time of data collection (2021/12/08–2022/08/14) were mainly in not peer-reviewed preprint form on bioRxiv, medRxiv or otherwise in the public domain (Supplementary Fig. S1). By now, many of these preprints have been published in peer-reviewed journals. However, we collected the data in real time and updated a publicly available Google Slide deck^2^, summarizing each study, and a publicly accessible Google Sheets document incrementally with incoming data^3^, also available in the manuscript’s GitHub repository (https://github.com/acorg/netzl_et_al2025/blob/main/data/google_sheet_tables/Netzl%20et%20al.%20-%20Collected%20Omicron%20antigenic%20data.csv). We base our analysis here on such collected data, the first publicly available preprints, in order to emulate the real-world scenario of a novel emerging variant and the urgency that comes with reporting its immune escape. To test that our analysis holds for published data, we randomly selected eight studies, corresponding to roughly 10% of the preprint data we used at the time, and compared the preprint-extracted data with the data given in the final publication (Supplementary Table S1). All values were consistent between preprint and publication except for a single study which added samples after BA.1 or BA.2 (breakthrough) infection that marginally changed reported GMTs. Importantly, we found no differences in the 2x and 3x Vax groups, which were the focus groups from a public health perspective at the time of BA.1 emergence. Given the consistency of preprinted and published data in this subset, and the substantial time-consuming nature of data extraction, we did not repeat the process for the remaining 90% of studies.

We collected data indiscriminate of academic institution or geographical location, but found a strong bias towards studies from the US and Germany in our final dataset, with more than half of all collected studies having a corresponding author located at a German or US institution (Table 1, Supplementary Fig. S2). Further, the majority of subjects across studies were female (Supplementary Fig. S3).

**Table 1:** List of included studies. An overview of the collected studies. The link to the document from where the data was extracted is given in^3^. The time lists the upload date of the study extracted from the source link, or when the data was added by the authors (DD/MM/YYYY). The studies were named after the first or the corresponding author, the corresponding author’s country affiliation is listed in the Country column. The neutralization assay virus type is given in the assay type column and refers to a specific pseudotype if not labeled “Pseudovirus” or “Authentic virus”. The cell line shows which cells were used in the neutralization assay. “N/A” indicates that no information was found. Results from the Pfizer/BioNTech study were reported in two different sources^49,50^ and hence shown in two rows. Studies which reported data from multiple assays are shown in a row per assay (n=5), resulting in a table with 90 rows for 85 different sources.

Study name	Time	Country	Assay type	Cell line
Pfizer/BioNTech^49^	08/12/2021	USA	Pseudovirus	N/A
Pfizer/BioNTech^50^	08/12/2021	USA	Pseudovirus	N/A
Sigal^51^	08/12/2021	South Africa, Germany	Authentic virus	H1299-ACE2
Snape^52^	10/12/2021	UK	Authentic virus	Vero
Poehlmann^53^	12/12/2021	Germany	VSV	Vero
Balazs^15^	14/12/2021	USA	Lentiviral	293T-ACE2
Schmidt^54^	12/12/2021	USA	HIV-1	HT1080/ACE2
Israel MoH^55^	12/12/2021	Israel	Authentic virus	N/A
HKU^56^	12/12/2021	China	Authentic virus	N/A
Gruell^57^	14/12/2021	Germany	Lentiviral	293T-ACE2
Ho^58^	14/12/2021	USA	VSV	Vero-E6
Ho^58^	14/12/2021	USA	Authentic virus	Vero TMPRSS2
Schwartz^59^	14/12/2021	France, Belgium	Authentic virus	S-Fuse
Montefiori/Doria-Rose^60^	15/12/2021	USA	Lentiviral	N/A
Montefiori/Doria-Rose^60^	15/12/2021	USA	Authentic virus	N/A
Gao^61^	16/12/2021	China	VSV	Vero
Chen^62^	17/12/2021	China	Lentiviral	293T-ACE2
Krammer^63^	20/12/2021	USA	Authentic virus	Vero-E6 TMPRSS2
Shi^64^	20/12/2021	USA	Authentic virus	Vero-E6
Suthar^18^	20/12/2021	USA	Authentic virus	Vero-E6 TMPRSS2
Cicin-Sain^65^	21/12/2021	Germany	VSV	Vero-E6
Screaton^7^	22/12/2021	UK	Authentic virus	Vero
Sahin^66^	22/12/2021	Germany	VSV	Vero76
Weiss^67^	22/12/2021	USA	Lentiviral	293T-ACE2/TMPRSS2
Haveri^68^	22/12/2021	Finland	Authentic virus	Vero-E6
Arien^69^	23/12/2021	Belgium	Authentic virus	Vero
Wang^70^	24/12/2021	China	VSV	Vero-E6
Takahashi^71^	24/12/2021	Japan	VSV	Vero-E6 TMPRSS2
Eckerle^72^	28/12/2021	Switzerland	Authentic virus	Vero-E6
Landau^73^	28/12/2021	USA	Lentiviral	293T-ACE2
Suzuki^74^	01/01/2022	Japan	Authentic virus	Vero-E6 TMPRSS2
Suzuki^74^	01/01/2022	Japan	VSV	Vero-E6 TMPRSS2
Barouch^75^	02/01/2022	USA	Lenti-CMV	293T-ACE2
Sanders^76^	03/01/2022	The Netherlands, USA	Lentiviral	293T-ACE2
Garg^77^	04/01/2022	India	Authentic virus	Vero-E6
Wang(CDC)^78^	05/01/2022	USA	Authentic virus	Vero-E6 TMPRSS2
Gintsburg^79^	15/01/2022	Russia	Authentic virus	Vero-E6
Münch^80^	17/01/2022	Germany	VSV	Vero-E6
Poovorawan^81^	17/01/2022	Thailand	Authentic virus	N/A
Mizukami^17^	20/01/2022	Japan	Authentic virus	Vero-E6 TMPRSS2
Xia/Swanson/Shi^82^	21/01/2022	USA	Pseudovirus	Vero-E6
Pajon/Doria-Rose^83^	26/01/2022	USA	Lentiviral	293T-ACE2
Protzer^84^	28/01/2022	Germany	Authentic virus	MDA-MB-231-hACE2
Medigeshi^85^	28/01/2022	India	Authentic virus	Vero-E6
Yu/Barouch^86^	06/02/2022	USA	Lenti-CMV	293T-ACE2
Iketani/Ho^87^	07/02/2022	USA	VSV	Vero-E6
Xie^88^	07/02/2022	China	VSV	Huh 7
Peiris/Cheng^89^	08/02/2022	China	Authentic virus	Vero-E6 TMPRSS2
Sato^90^	14/02/2022	Japan	HIV-1	HOS-ACE2/TMPRSS2
Mykytyn/Haagmans^91^	23/02/2022	The Netherlands	VSV	Vero-E6
Suzuki/Kawaoka^92^	24/02/2022	USA	Authentic virus	Vero-E6 TMPRSS2
Veesler/Bowen^93^	15/03/2022	USA	VSV	Vero-E6 TMPRSS2
Shi/Biontech^94^	24/03/2022	USA	Spike	Vero-E6
Moore^95^	25/03/2022	South Africa	Lentiviral	293T-ACE2
To^96^	28/03/2022	China	Authentic virus	Vero-E6 TMPRSS2
Krumbholz^97^	29/03/2022	Germany	N/A	N/A
Shi2^98^	30/03/2022	USA	Spike	Vero
Thalin^99^	02/04/2022	Sweden	Authentic virus	Vero-E6
Klein^100^	06/04/2022	Germany	Lentiviral	293T-ACE2
Hoffman^101^	12/04/2022	Germany	Pseudovirus	Vero
Stiasny^102^	13/04/2022	Austria	Authentic virus	Vero-E6
Liu/Evans^103^	15/04/2022	USA	Lentiviral	293T-ACE2
Sigal/Khan2^104^	29/04/2022	South Africa, Germany	Authentic virus	H1299-E3
Xie/Cao^105^	30/04/2022	China	VSV	293T
Wang2^106^	03/05/2022	China	Pseudovirus	Vero
Poehlmann2^107^	05/05/2022	Germany	Pseudovirus	Vero
Sigal/Khan^108^	06/05/2022	South Africa, Germany	Authentic virus	H1299-E3
Chen/Zhou^16^	09/05/2022	China	HIV-1	293T-ACE2
Kimpel/Roessler^45^	10/05/2022	Austria	Authentic virus	Vero ACE2/TMPRSS2
Liu2^109^	16/05/2022	USA	Lentiviral	293T
Barouch2^110^	16/05/2022	USA	Pseudovirus	293T
Barclay^111^	20/05/2022	UK	Lentiviral	293T-ACE2
Screaton^38^	21/05/2022	UK	Lentiviral	293T/17-ACE2
Peacock/Doores^112^	25/05/2022	UK	Lentiviral	293T-ACE2
Ho/Liu^113^	26/05/2022	USA	VSV	Vero-E6
Weiss/Wang^34^	01/06/2022	USA	Lentiviral	293T-ACE2/TMPRSS2
Kurhade/Shi^94^	05/06/2022	USA	Authentic virus	Vero-E6
Weiss/Wang2^114^	05/07/2022	USA	Lentiviral	293T-ACE2/TMPRSS2
Turville^115^	07/07/2022	Australia	Authentic virus	HAT-24
Wang/Cao^116^	18/07/2022	China	VSV	Huh 7
Murrel^117^	19/07/2022	Sweden	N/A	293T-ACE2
Liu/Qu^118^	21/07/2022	USA	Lentiviral	293T-ACE2
Shi/Xie^119^	29/07/2022	USA	Authentic virus	Vero-E6
Ho/Wang^120^	31/07/2022	USA	VSV	Vero-E6
Sahin/Muik^121^	02/08/2022	Germany	VSV	Vero76
Sahin/Muik^121^	02/08/2022	Germany	Authentic virus	Vero-E6
Wang/Wang^122^	04/08/2022	China	VSV	Vero-E6
Klein/Gruell^123^	04/08/2022	Germany	Lentiviral	293T-ACE2
Liu/Qu2^124^	14/08/2022	USA	Lentiviral	293T-ACE2

The collected data contained neutralization data of various Omicron sublineages (B.1.1.529: BA.1 and BA.1+R346K (BA.1.1); BA.2, BA.2.12.1 and BA.2.75; BA.3; BA.4/5) as well as ancestral and other SARS-CoV-2 variants by different vaccine sera and sera of individuals infected with the ancestral virus (614D/G, from here onwards referred to as wild type WT), Alpha (B.1.1.7), Beta (B.1.351), Gamma (P.1) or Delta (B.1.617.2) variant. As time progressed, other Omicron lineages emerged, and we kept collecting the neutralization data as it was generated (Supplementary Fig. S1). In this study, however, we did not include neutralization data from Omicron sublineages later than BA.2. For the purpose of the current study, to assess the usefulness of collating public data from variable sources for an early assessment of a variant’s immune escape, we focused our analyses on the BA.1 lineage. BA.1 was the first Omicron variant to circulate, and therefore the sublineage for which we extracted the most data, allowing us to draw more meaningful conclusions on the impact of variability of public data.

The majority of the collected data was generated using the Omicron BA.1 lineage. Some research groups indicated that the virus they used had the R346K mutation (BA.1.1, BA.1+R346K). To identify whether this substitution impacted neutralization, we compared BA.1 and BA.1.1 neutralization titers in the same sera and found no statistically significant difference of GMTs for the two lineages (Supplementary Fig. S4, Supplementary Table S2).

As the data was generated in different laboratories with little coordination between laboratories, a variety of neutralization assays and cell types was used, an overview is given in Table 1. We categorized the serum panels used by the different laboratories by their infection or vaccination history into different serum groups as described in the methods section.

### Collective data can inform public health decisions early after variant emergence

**Fig. 1:**
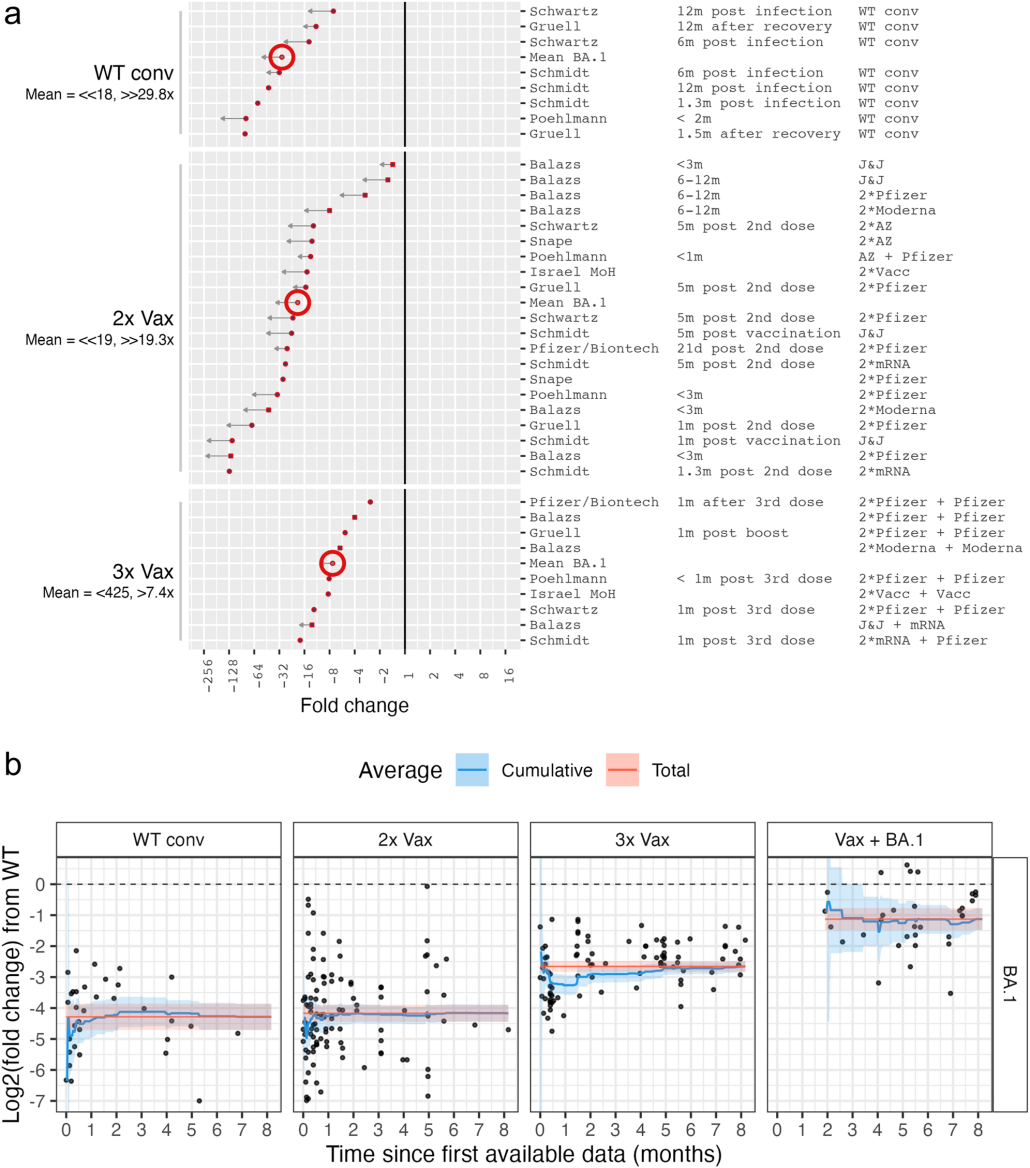
BA.1 neutralization titer fold change from WT (614D/G) titers over time. **(a)** Fold changes of GMT neutralization titers as reported by individual studies are shown for the first two weeks of data reporting (n=10 studies), ordered by magnitude and stratified by serum group. Row labels show the study name (Table 1), time since exposure and serum type. The big red circle indicates the mean value for each group. Below the serum group label, the BA.1 geometric mean titer (GMT) is given followed by the mean fold change from WT titers. Arrows in the plot and “</>” in the label indicate uncertainties in the point estimate due to titers below the limit of detection (LOD) of the assay. A short arrow (“>”/”<”) marks measurements with more than half of BA.1 titers below the assay’s LOD, or conversely reference antigen titers at or lower than the LOD. Long arrows (“>>”/”<<”) mark measurements with more than approximately 80% of BA.1 titers below the LOD. The solid vertical line marks no fold change. (**b)** Log2 fold changes from WT GMTs to BA.1 GMTs are shown for individual studies by time since the first reported data (1 month = 30 days). The blue line shows the cumulative mean with 95% CI indicated by the shaded area, the red line with shaded area corresponds to the mean fold change with 95% CI at the end of the data collection period. The data is stratified by exposure history.

Early in the pandemic, neutralization titers were established as correlates of protection against severe disease^11,12^. Therefore, measures such as breadth of neutralization across variants and fold reduction of titers were used to inform public health decisions, including vaccine strain updates after the emergence of the BA.1 variant^13,14^. Data from government approved laboratories and clinical trials using standardized assays contribute hugely to informing these decisions but take time to generate. At the same time, individual studies generate these data quickly, but in a less controlled manner. To assess BA.1’s immune escape from WT, we extracted the reported mean fold changes or calculated fold changes from reported geometric mean titers (GMTs) of individual studies in different serum cohorts. We did this incrementally, adding new data in real time and summarizing it in public documents^2^, and we show data within the first two weeks of reporting in Fig. 1a.

We continued collating data and were interested how soon after variant emergence enough data was available to obtain stable immune escape estimates relevant for public health guidance. To do so, we examined how the mean neutralization titer fold drop from WT to BA.1 changes over time with the addition of more data (Fig. 1b). For WT conv (614D/G convalescent) and 2x Vax (twice vaccinated), there was already enough data reported within the first 15 days of data reporting such that the cumulative mean was within the 95% CI (confidence interval) of the total mean and remained stable over the remaining collection period. In the 3x Vax group, the fold change from WT was overestimated by one two-fold between two and six weeks after data reporting and converged to the total mean thereafter.

We calculated the confidence intervals of the cumulative means as a measure of how reliable the mean estimates are up to a given time point. We found that 2 weeks of data collection for the WT conv, 2x Vax and 3x Vax group was sufficient to bring the cumulative mean 95% CI of all three groups to a single log2 unit precision. After a month of data collection it was further improved up to half a log2 unit for the Vax groups. The cumulative mean 95% CI for the Vax + BA.1 group was generally wider throughout data collection, especially early on, owing to less data availability for this type of sera. Nevertheless, the 95% CI for the Vax + BA.1 was less than two log2 units one month after the first Vax + BA.1 data was collected, and less than one and a half log2 units after a month and a half.

In addition, we found clear evidence of the benefit of a third vaccine dose over two doses already after two weeks, which only became more certain over time. Further, we found that an exposure to BA.1 after vaccination greatly reduces BA.1’s immune escape. This observation was only possible once enough BA.1 breakthrough infections occurred to be assayed. Despite this lag in reporting, employing public data indicated the benefit of a vaccine strain update to BA.1 already 4 months after its emergence. These observations are further explored in section “Antigenic cartography of BA.1’s immune escape” via use of antibody landscapes.

To get a confidence estimate of the variance in the titer fold change calculation after, say, ten studies we randomly selected (with replacement) ten of all samples of 2x Vax data. We repeated this random selection (bootstrapping) 11 times, corresponding to 10% of the number of 2x Vax samples, and calculated the mean WT to BA.1 fold change each time. We next calculated the 95% CI of this distribution of mean fold changes and repeated this bootstrap process for all number of samples from 1 to 108, and additionally for the 3x Vax data (Supplementary Fig. S5). We found that with just 10 randomly selected samples, the overall fold change was within the 95% CI of the mean fold changes for both the 2x and 3x Vax cohorts. With 20 and 15 random samples, respectively, the range of lower to upper 95% CI bound was lower than 1.5x, meaning that both would be within one dilution step of a neutralization assay, and within the population’s mean 95% CI.

### Omicron BA.1’s immune escape from wild type in different exposure histories

**Fig. 2:**
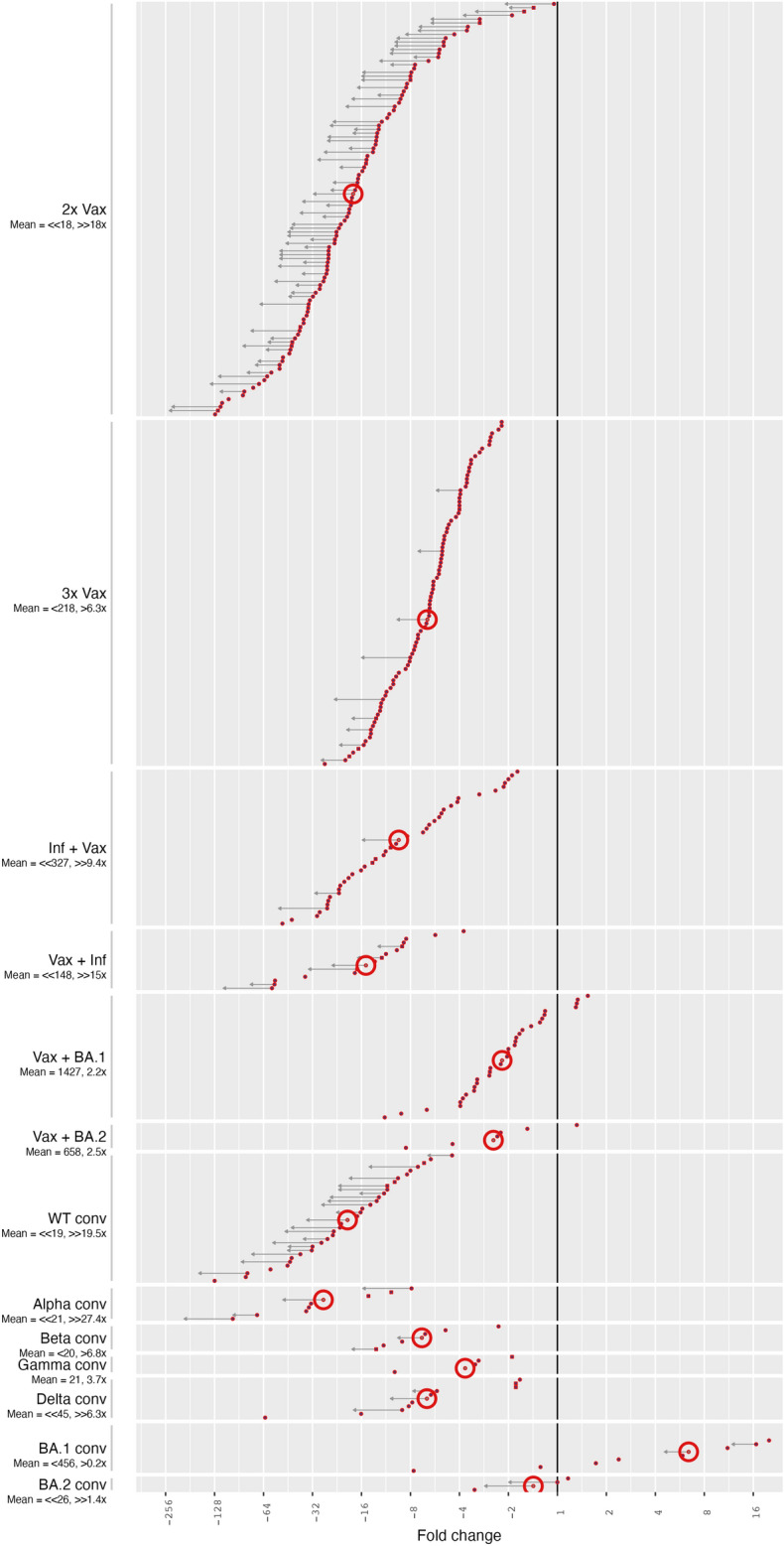
Omicron BA.1 neutralization titer fold changes relative to WT (614D/G). Same as Fig. 1a but without row labels.

In the same style as Fig. 1a, we present the BA.1 fold drops from WT at the final stage of data collection in Fig. 2 for more types of exposure histories and more laboratories for exposure groups that already exist in Fig. 1a. The numerical data in all serum groups and for other variants is summarized in Tables 2,3,4.

**Table 2:** Variant Geometric Mean Titers (GMT) per serum group (conv = convalescent). The 95% CI is given in parentheses, the number of data points n in the next line. WT summarizes wild type-like strains (e.g: 614D, 614G).

Serum Group	WT	Alpha	Beta	Gamma	Delta	BA.1
2x Vax	334 (253; 440)	173 (70; 426)	75 (53; 105)	153 (43; 541)	123 (81; 185)	18 (15; 22)
	n=87	n=10	n=40	n=6	n=44	n=87
3x Vax	1344 (1014; 1781)	453 (200; 1026)	354 (210; 598)		398 (245; 648)	221 (163; 300)
	n=82	n=9	n=27		n=32	n=82
Inf + Vax	2840 (1621; 4977)	683	661 (250; 1743)	360	1677 (851; 3305)	348 (182; 665)
	n=31	n=1	n=9	n=1	n=16	n=31
Vax + Inf	3353 (1281; 8775)	2223 (294; 16813)	1090 (34; 34774)	1112	2100 (761; 5792)	190 (51; 708)
	n=10	n=2	n=3	n=1	n=10	n=10
Vax + BA.1	3272 (2039; 5250)	807 (120; 5434)	1120 (427; 2942)		942 (467; 1903)	1505 (977; 2319)
	n=31	n=5	n=10		n=17	n=31
Vax + BA.2	1626 (699; 3782)	1486 (1302; 1697)	547 (377; 793)		668 (265; 1683)	658 (193; 2240)
	n=6	n=2	n=2		n=3	n=6
WT conv	405 (246; 667)	176 (30; 1044)	56 (17; 184)	37 (2; 565)	183 (89; 376)	18 (13; 27)
	n=29	n=6	n=7	n=3	n=16	n=29
Alpha conv	668 (65; 6815)	699 (58; 8501)	142 (12; 1679)	66 (0; 29728)	328 (23; 4763)	23 (7; 84)
	n=6	n=5	n=5	n=3	n=6	n=6
Beta conv	110 (9; 1290)	133 (2; 10303)	298 (11; 7976)	158 (0; 596407)	75 (10; 589)	27 (3; 215)
	n=5	n=4	n=4	n=3	n=5	n=5
Gamma conv	67 (4; 1086)	77 (2; 3341)	103 (2; 5508)	275 (0; 179386178094)	26 (0; 1470)	20 (1; 466)
	n=3	n=3	n=3	n=2	n=3	n=3
Delta conv	437 (81; 2371)	195 (14; 2706)	72 (5; 1090)	70 (3; 1939)	2294 (451; 11673)	50 (14; 185)
	n=8	n=5	n=5	n=3	n=8	n=8
BA.1 conv	71 (26; 194)	40 (3; 460)	62 (19; 205)	16 (0; 31041577)	53 (17; 167)	456 (144; 1438)
	n=12	n=4	n=7	n=2	n=10	n=12
BA.2 conv	37 (0; 6933)	11	11	5	15 (0; 3013)	26 (1; 840)
	n=3	n=1	n=1	n=1	n=2	n=3

**Table 3:** BA.1 titer fold changes from variants per serum group (conv = convalescent). Mean fold drops were calculated from fold drops per study, not from GMT fold drops. Studies reporting fold drops but not GMTs result in a discrepancy between GMT based mean fold drop and individual study based mean fold drop (Table 2). The 95% CI is given in parentheses, the number of data points n in the next line. WT summarizes wild type-like strains (e.g: 614D, 614G).

Serum Group	WT	Alpha	Beta	Gamma	Delta
2x Vax	18 (14.9; 21.7)	14.6 (6.3; 34.2)	4.2 (3.3; 5.3)	18.2 (8.4; 39.8)	6.8 (5.2; 8.8)
	n=108	n=11	n=44	n=7	n=51
3x Vax	6.3 (5.6; 7.1)	4 (3.4; 4.6)	2.6 (2.1; 3.1)		2.9 (2.4; 3.5)
	n=90	n=9	n=30		n=34
Inf + Vax	9.4 (7; 12.8)	10.5	5.9 (3.7; 9.3)	5.5	4.7 (2.5; 8.9)
	n=40	n=1	n=12	n=1	n=16
Vax + Inf	15 (9.4; 24)	15.1 (0.9; 242.4)	3 (1.7; 5.4)	11	8.4 (4.5; 15.7)
	n=15	n=2	n=3	n=1	n=12
Vax + BA.1	2.2 (1.7; 2.8)	1 (0.4; 2.9)	1 (0.7; 1.5)		1.3 (0.8; 2.1)
	n=32	n=5	n=10		n=18
Vax + BA.2	2.5 (1; 6)	3.8 (0.8; 17.4)	1.4 (0.5; 3.9)		1.8 (0.7; 4.1)
	n=6	n=2	n=2		n=3
WT conv	19.5 (14.6; 26.1)	13.1 (7.3; 23.6)	4.1 (2; 8.1)	6.8 (2.5; 18.8)	10.6 (5.7; 19.7)
	n=33	n=7	n=9	n=4	n=18
Alpha conv	27.4 (13; 57.7)	43.2 (21.1; 88.3)	10.2 (7.6; 13.8)	9.7 (4.2; 22.4)	14.9 (7.3; 30.6)
	n=8	n=7	n=6	n=4	n=8
Beta conv	6.8 (3.5; 13.4)	11 (3.9; 31.5)	24.7 (15.2; 40.3)	18.1 (2.5; 131.8)	4.4 (1.4; 13.8)
	n=6	n=5	n=5	n=4	n=6
Gamma conv	3.7 (1.2; 11.3)	4.2 (1.9; 9.2)	6.1 (3.6; 10.3)	15.1 (2; 112.7)	1.4 (0.9; 2.3)
	n=4	n=4	n=4	n=3	n=4
Delta conv	6.3 (2.8; 14.1)	6 (2; 18.1)	2.5 (0.5; 11.8)	3.2 (0.4; 28.9)	36.7 (19.4; 69.4)
	n=10	n=6	n=6	n=4	n=10
BA.1 conv	0.2 (0.1; 0.5)	0.1 (0; 0.9)	0.2 (0; 0.6)	0.1 (0; 0.7)	0.2 (0.1; 0.5)
	n=12	n=4	n=7	n=2	n=10
BA.2 conv	1.4 (0.2; 8.5)	0.8	0.8	0.4	1.3 (0.1; 30)
	n=3	n=1	n=1	n=1	n=2

**Table 4:** Variant titer fold changes from WT (614D/G) per serum group (conv = convalescent). Mean fold drops were calculated from fold drops per study, not from GMT fold drops. Studies reporting fold drops but not GMTs result in a discrepancy between GMT based mean fold drop and individual study based mean fold drop (Table 2). The 95% CI is given in parentheses, the number of data points n in the next line. WT summarizes wild type-like strains (e.g: 614D, 614G).

Serum Group	Alpha	Beta	Gamma	Delta
2x Vax	1.3 (1; 1.7)	4.6 (3.8; 5.5)	2.6 (1.6; 4.2)	2.7 (2.3; 3.3)
	n=11	n=44	n=7	n=50
3x Vax	1 (0.7; 1.4)	2.6 (2.1; 3.3)		2.3 (1.9; 2.6)
	n=9	n=30		n=33
Inf + Vax	1.7	3.1 (2.2; 4.2)	3.3	1.8 (1.2; 2.8)
	n=1	n=12	n=1	n=16
Vax + Inf	1.2 (0; 504.7)	5 (1.3; 18.9)	3.2	1.9 (1.2; 2.9)
	n=2	n=3	n=1	n=10
Vax + BA.1	1.1 (0.5; 2.3)	2.2 (1.4; 3.7)		1.8 (1.3; 2.5)
	n=5	n=10		n=15
Vax + BA.2	0.7 (0.7; 0.7)	1.7 (1.7; 1.7)		2.1 (0.1; 30)
	n=2	n=2		n=3
WT conv	2.1 (1.5; 3)	6.1 (3.7; 10)	3.5 (1.2; 10.1)	2.2 (1.7; 2.8)
	n=7	n=9	n=4	n=18
Alpha conv	0.6 (0.3; 1)	2.7 (1; 7.4)	2.6 (0.4; 15.4)	1.8 (1.2; 2.8)
	n=7	n=6	n=4	n=8
Beta conv	0.8 (0.3; 2.3)	0.3 (0.2; 0.8)	0.4 (0.1; 3.2)	1.5 (0.6; 3.9)
	n=5	n=5	n=4	n=6
Gamma conv	0.9 (0.5; 1.6)	0.6 (0.3; 1.2)	0.3 (0.1; 0.9)	2.6 (0.9; 7.6)
	n=4	n=4	n=3	n=4
Delta conv	1.1 (0.7; 1.7)	2.7 (1.4; 5)	1.6 (0.3; 7.8)	0.2 (0.1; 0.3)
	n=6	n=6	n=4	n=10
BA.1 conv	1.7 (1.1; 2.5)	1.5 (0.8; 2.7)	1 (0; 70)	1.1 (0.8; 1.7)
	n=4	n=7	n=2	n=9
BA.2 conv	1.1	1.1	2.4	0.7 (0; 45.1)
	n=1	n=1	n=1	n=2

The double and triple vaccinated serum groups constituted the majority of the data that have been reported and consequently analyzed here and were the most relevant from a public health perspective at the time of data collection. The 2x Vax serum group contained the highest number of individual measurements and exhibited the widest spread and largest uncertainty in fold drops of BA.1 neutralization compared to WT. The 2x Vax group showed the most variability in fold changes from WT, which we attribute to a wide range of reported WT titers, serum collection times from two weeks to nine months post second dose, and limit of detection (LOD) censoring of low to non-detectable Omicron titers (Supplementary Fig. S6-S7). Time since exposure impacts titer magnitude and measured fold changes if the waning across variants over time is non-homogeneous, as we discuss later in section “Increased cross-neutralization over time since exposure”. Neutralization titers measured around the peak of immune responses, between two and 4 weeks post exposure, were highly variable in the collected data (Supplementary Fig. S7).

We found an average fold drop of 18x in this serum group (Fig. 2, Table 3) when treating measurements below an assay’s LOD in the common manner as LOD/2. However, the majority of fold changes were likely greater than the point estimates due to many BA.1 titers being below the LOD. Consequently, the average fold drop is likely substantially greater than 18x. We found that in many studies with an unexpectedly low fold drop from WT to BA.1 titers, titers against WT were very low^15–18^. Low titers against the reference antigen limit the amount of further reduction until an assay’s detection limit is reached, resulting in LOD censoring of titers and seemingly low fold drops (Supplementary Fig. S6-S7).

LOD censoring did not occur in the 3x Vax group where all WT and almost all BA.1 titers were detectable (Supplementary Fig. S6). Hence, the estimated average fold drop of 6.3x for this group is more reliable compared to the 2x Vax group and demonstrates the benefit of a third WT vaccination, which was much debated at the time of BA.1’s emergence. We investigated if the substantially lower fold drop in 3x Vax compared to 2x Vax is because higher titers in 3x Vax were underreported, either by laboratories not titrating to the endpoint, or because of a high-titer non-linearity in the assay by looking at titers and reported fold drops and found that the fold drop from WT to BA.1 was independent of titer magnitude against WT (Supplementary Fig. S6). A third vaccine dose also reduced fold changes more than non-Omicron breakthrough infections, with fold drops from WT of 15x and 9.4x in the Vax + Inf and Inf + Vax groups. Additionally, a third dose lifted titers against BA.1 above a level identified to be protective against symptomatic infection during WT circulation (Supplementary Fig. S6).

The effect of low WT titers on fold drops from WT to BA.1 is also seen in convalescent serum cohorts, despite the limited amount of data for most of these cohorts. While high titers against WT after infection with a WT-like variant resulted in mean fold drops from WT of 19.5x and 27.4x in the WT and Alpha conv (convalescent), respectively, mean fold drops in Beta and Delta conv were much lower, at 6.8x and 6.3x due to lower WT titers (Supplementary Fig. S7, Table 2-3). On the other hand, BA.1 fold drops relative to the infecting Beta and Delta variants were much higher compared to BA.1 fold drops from WT for the same groups: 24.7x and 36.7x (vs 6.8x and 6.3x) (Table 3). One possible mechanism is again an LOD effect reducing fold drops from WT in Beta and Delta convalescent serum groups, since these groups will have higher homologous titers compared to WT titers. Another possible mechanism could be neutralizing antibodies in Beta and Delta sera focusing on regions that are distinct from both WT and BA.1 and therefore, with respect to these sera, WT and BA.1 appear antigenically less distinct leading to lower titer differences between these antigens.

As expected, a (breakthrough) infection with either BA.1 or BA.2 substantially increased titers against BA.1, at times even above WT titer levels, and resulted in average fold drops smaller than 3x.

In general, we saw high variability of fold drop data within all serum groups in the whole dataset, likely owing to several factors such as age of participants, serum collection times, and different assays and cell types used to assess serum neutralization ability (Table 1). For the 3x Vax and WT conv groups, we found a statistically significant difference between fold changes measured using authentic virus (LV) and pseudovirus (PV), but while LV fold changes were significantly higher than PV in 3x Vax it was the other way around in WT conv (Supplementary Table S3, Supplementary Fig. S7). For reported GMTs however, we consistently found higher PV than LV GMTs, often at a significant level (Supplementary Tables S5-S6, Supplementary Fig. S7). Different serum collection times can affect fold drops either through an LOD mechanism as explained above or through higher cross-reactivity as demonstrated in Wilks et al.^19^. Still, combining data from various sources gives quick and reliable results as shown in Fig. 2, and a public database where laboratories enter their results would greatly contribute to quick decision making, and further, assay refinement across laboratories.

### Antigenic cartography of BA.1’s immune escape

As an additional way to evaluate the reliability of variable source data for immunological surveillance, we constructed antigenic maps from the collected data and compared them to single source antigenic maps. In an antigenic map, virus variants are positioned based on their antigenic properties inferred by fold drops in serum neutralization titers^20^. Variants that elicit similar titers in the same sera are positioned at small distances from each other, and vice versa. Antigenic maps are a key instrument for vaccine strain selection for influenza vaccines and have also been used to investigate SARS-CoV-2 vaccine strain updates^21^.

**Fig. 3:**
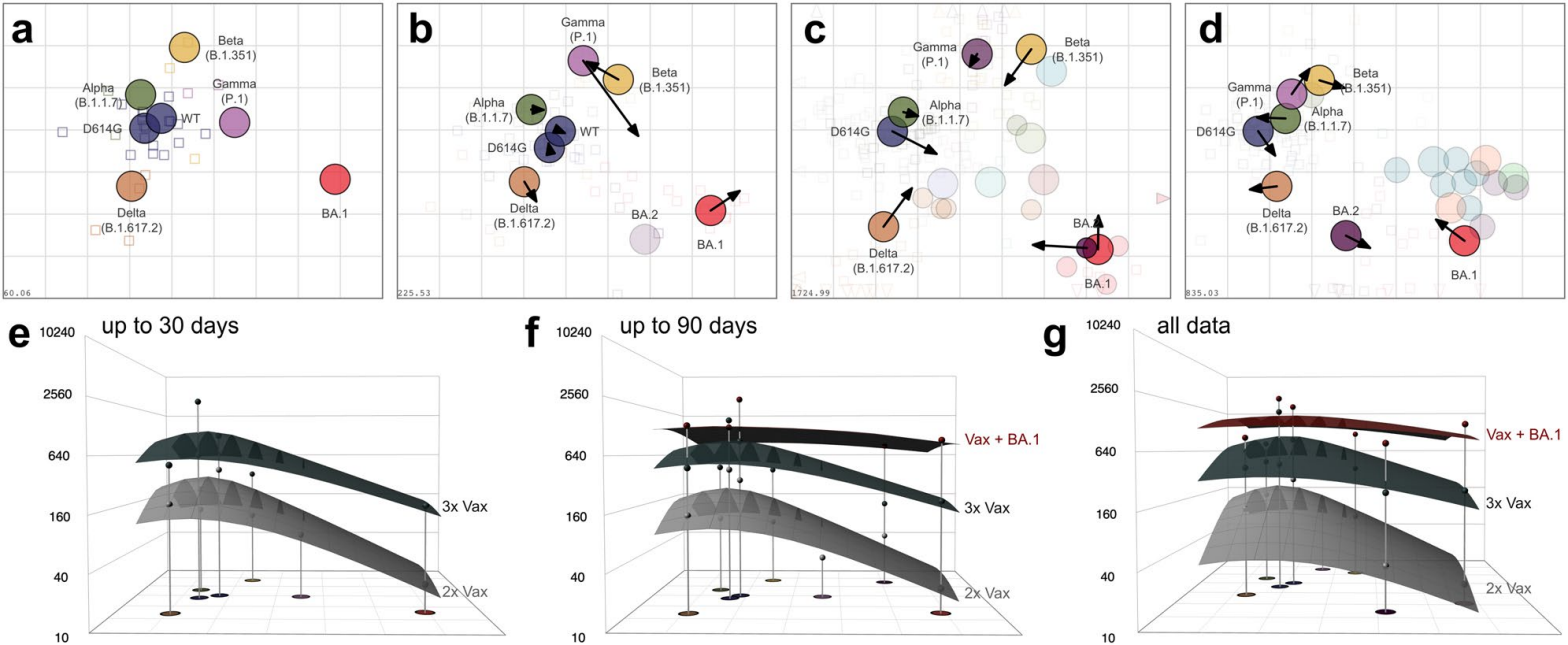
Antigenic cartography. (**a**) An antigenic map from convalescent data up to 1 month post first available data was constructed. Variants are shown as labeled, colored circles and sera are shown as open squares with the color matching the infecting variant. The x- and y-axes correspond to relative antigenic distances, each grid line reflects an additional 2-fold dilution in the neutralization assay. (**b**) An antigenic map constructed from all convalescent data with arrows pointing to the variants’ positions in a. (**c**) Comparison of the map by Wilks et al.^19^. to the full data map in b and (**d**) the map by Roessler et al.^22^. to the full data map in b. Arrows point to the variants’ positions in the respective map. The numbers on the bottom left corner show the stress of the map. (**e**-**f**) GMT antibody landscapes for the 2x Vax (grey), 3x Vax (dark grey) and Vax + BA.1 (red) serum groups are shown, subset to early data up to 1 month post first report (**e**), medium data up to 90 days post first report (**f**) and all data (**g**). GMTs against variants are indicated by impulses.

Using data only from convalescent serum cohorts, we again found that data within one month of reporting produced a very stable result (Fig.3 a-b) (Supplementary Fig. S8-S13). Only Gamma’s position changed slightly when creating a map from all data (Fig. 3b). Already after one month of data reporting, the map captured BA.1’s complete escape from all pre-Omicron sera and variants. The early data map and the full data map were highly consistent with maps from single laboratories, both a map constructed using pseudovirus neutralization data^19^ (Fig. 3c) and one using authentic virus^22^ (Fig. 3d). Maps constructed using only authentic virus or pseudovirus neutralization data resulted in very similar variant positions for variants with sufficient titrations in the different serum groups (Supplementary Fig. S8). We found that the authentic virus map is marginally condensed compared to the pseudovirus map, visible by arrows pointing outwards in Supplementary Fig. S8. This is in line with the on average lower fold drops reported in authentic virus assays in single exposure cohorts (Supplementary Tables S3-S4).

Whereas antigenic maps show individual sera as points based on their reactivity, antibody landscapes show the distribution of neutralization titers for individual sera against multiple strains as a surface in a third dimension above an antigenic map^23^. To illustrate the effect of a third vaccination or breakthrough infection, we constructed antibody landscapes for the 2x Vax, 3x Vax and Vax + BA.1 serum groups, again for different end points (Fig. 3e-g). As before, we found that combining data from different sources gives stable representations of population immunity early after variant emergence. Moreover, we found a much heightened and broadened neutralizing response after, firstly, a third Wu-1 dose compared to only two doses, and secondly, BA.1 breakthrough compared to a third Wu-1 exposure. A third dose was necessary to lift BA.1 titers to a somewhat protective level compared to two doses (Supplementary Fig. S6).

### Increased cross-neutralization over time since exposure

**Fig. 4:**
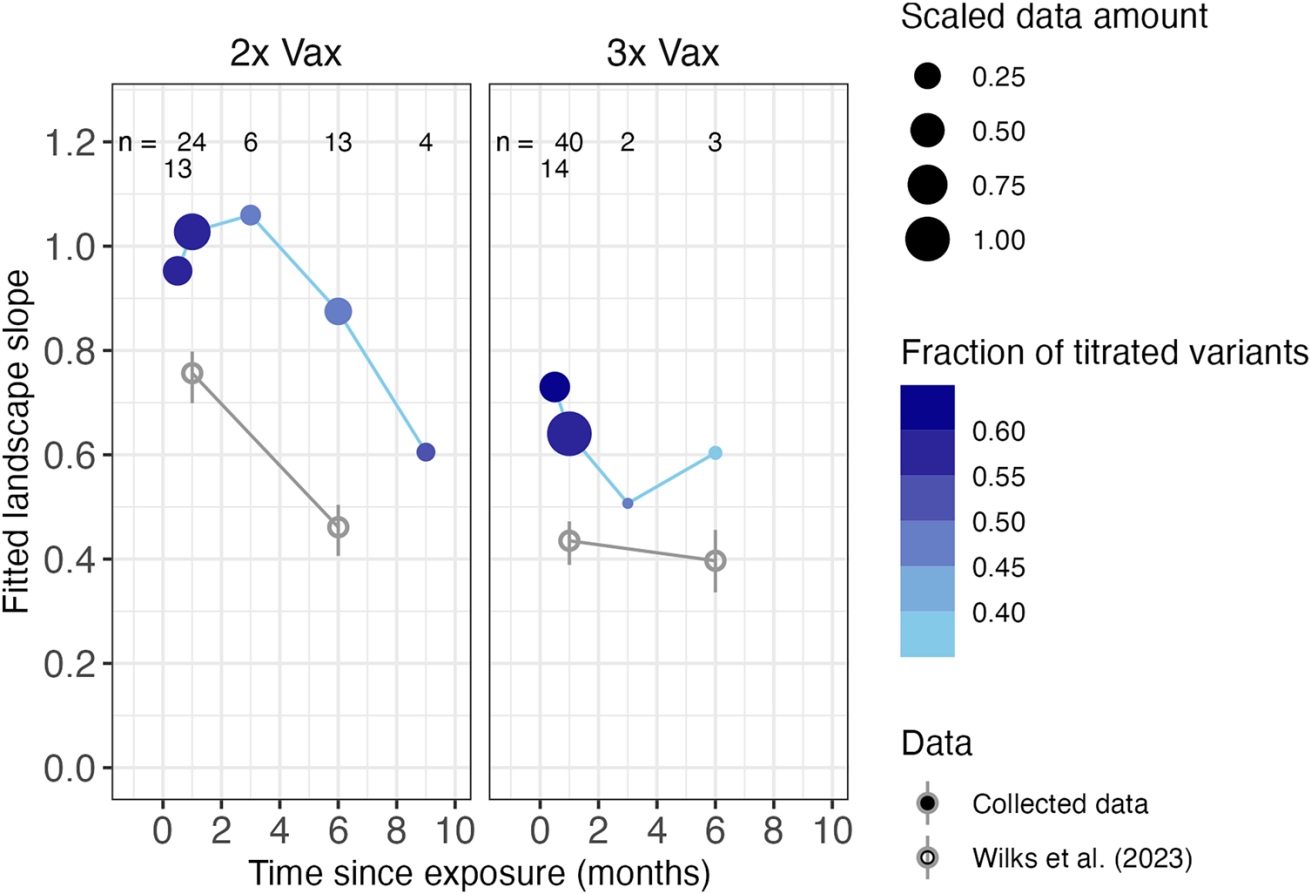
Antibody landscape slopes since exposure. Antibody landscapes were fitted to sera binned by their time since exposure for the 2x and 3x Vax cohorts. The time since exposure is given on the x-axis, the y-axis shows the fitted landscape slope. A low slope is indicative of broad cross-neutralization. The numbers in the top row indicate the number of sera that contributed to each landscape. The color of the point shows the fraction of variant titer measurements that contributed to the landscape fit, with a darker color indicating a more complete data set and better geometrical information for individual sera. The dot size gives the amount of data (n × fraction of titrated variants) per point scaled by the maximum data amount across serum cohorts. A detailed description is given in the methods section. The slopes reported by Wilks et al.^19^. are shown in grey and not scaled by data or fraction of titrated variants.

Antibody responses are highly dynamic processes, hence the timing of sampling impacts neutralization titer magnitude and fold changes. If sampled too early since exposure, immune responses did not have enough time to fully mature, resulting in generally low neutralization titers and seemingly broad cross-neutralization. After the peak immune response, titers gradually wane. Wilks et al.^19^, however, found that variant cross-neutralization increased over time after the second vaccination, and that cross-reactive antibodies were recalled after the third dose rather than induced by the third dose, indicating ongoing affinity maturation after the second dose. Following their approach, we constructed antibody landscapes for serum cohorts by time since exposure when the information was available. We binned individual sera by 2 weeks, 1, 3, 6, 9 and 12 months post exposure and fitted the landscape slope for these binned cohorts, with a smaller slope indicative of more cross-neutralization across antigenic space (Fig. 4).

We found an overall trend of lower slopes as time since exposure increased, indicating higher cross-neutralization. In general, the data with information since exposure was scarce and binning resulted in additional inaccuracies, explaining incoherences of individual data points. Still, the slope value 9 months after the 2^nd^ dose is remarkably similar to the slope values half a month and one month after the 3^rd^ dose, supporting the hypothesis by Wilks et al.^19^ that a third dose recalls broadened immunity rather than induces substantial broadening.

## Discussion

Quick actions in response to the emergence of pandemic-causing pathogens are essential to minimize the disease’s toll on human lives and the global economy. When Omicron BA.1 started to circulate in late 2021, its accumulation of known escape substitutions in the spike protein^24^ raised the concern that the Wu-1 targeted vaccine could be inefficient at protecting against BA.1, and that another lockdown might be necessary to prevent numerous hospitalizations and deaths. To provide decision makers and scientific advisory boards, such as the US NIH NIAID SAVE (SARS-CoV-2 Assessment of Viral Evolution), the UKHSA SAGE (UK Health Security Agency Scientific Advisory Group for Emergencies) and the WHO with the necessary information to recommend measures, we summarized publicly available data from preprints and publications from December 9, 2021 to August 14, 2022. During this time period, continuously updated versions of the here presented data were repeatedly presented and contributed to official advisory documents^2,8–10^. Here, we presented a more extensive analysis of this data and demonstrated the immense utility of rapidly, and readily, available antigenic data for public health and pandemic responses. Although many studies have shown a strong correlation of spike-protein binding antibodies and neutralization^25–27^, and Earle et al. found a stronger relationship between binding and vaccine efficacy than neutralization and efficacy^28^, we focused our analysis on neutralization titers, which were established as correlates of protection early in the pandemic and contributed to informing SARS-CoV-2 vaccine compositions^11–14^.

In response to one of the most urgent questions in late 2021, whether the recommendation of a third Wu-1 vaccine applies during BA.1 circulation, some definitive statements could be made from the aggregated neutralization data. Sera from individuals who had been vaccinated twice or infected with WT once showed generally more than an 18x fold drop of titers from WT, whereas people who had been vaccinated three times showed average fold drops of 6.3x (Table 3). Moreover, a third dose raised titers above a level reported to be protective against WT symptomatic infection (Supplementary Fig. S6). The reduced titer drop in triple vaccinated individuals appears to be real and not an artifact of the assay’s limit of detection, as fold drops were consistent over a range of titers. Censored titers below an assay’s detection threshold can result in a deflation of fold drops when titers against the reference antigen are low. The mean fold drop for the 2x Vax group, for example, is likely substantially greater than our numeric estimate. These results highlight the need for a more sophisticated analytical approach to treat values below an assay’s dynamic range than substituting them with a fixed value equal to half the detection threshold.

Remarkably, though, is that the above information was already in the public domain two weeks after BA.1’s emergence and remained largely stable thereafter (Fig. 2). Already after two weeks or ten random studies (Supplementary Fig. S5), we could identify the benefit of a third dose, albeit with less certainty. We show that even summary data, such as the geometric mean titers, contain immensely valuable information when combined from different sources. This real-world information could help massively in designing vaccination strategies and clinical trials, saving time and financial resources by more targeted design. As an example, antigenic cartography was used in the design of the COVAIL trial to investigate SARS-CoV-2 vaccine strain updates^21^. Antigenic cartography from the collected data did not only give a robust representation of antigenic relationships between pre-Omicron and BA.1 and BA.2 variants, but also showed the advantage of distant exposures through BA.1 breakthrough infection in broadening immunity (Fig. 3), which was described in independent studies and the COVAIL trial^19,21,29^.

Further, this analysis demonstrated that some of the phenomena observed in individual, more controlled, studies can also be seen in less controlled collated data. Wilks et al.^19^ described an increase in cross-reactivity after the second vaccination over time, and the collated data corroborate this (Fig. 4). The lower fold drops and higher titers after a third vaccine dose could be the result of ongoing affinity maturation in germinal centers after a second vaccination, and a recall of affinity matured B cells upon the third dose. Kim et al.^30^ reported the persistence of germinal centers for more than six months after mRNA vaccination, and found that antibodies derived from plasma cells with high levels of somatic hypermutation exhibited higher neutralization capacity against the D614G variant. Sokal et al.^31^ found that memory B cell-derived monoclonal antibodies of vaccinated individuals could maintain binding capacity to the Beta variant during affinity maturation. This, in combination with broader antibody landscapes after the third dose, suggests that repeated vaccination with the original spike protein has the potential to boost cross-reactive immunity across antigenic space.

Finally, data from different sources provide valuable information for assay standardization. We found a statistically significant difference between pseudovirus- and authentic virus-assessed fold drops from WT to BA.1 in the WT conv and 3x Vax group. The collected data was generated using a variety of pseudotypes and cells, with different ACE2 and TMPRSS2 expression patterns. SARS-CoV-2 evolved in ways that elicited differential usage of TMPRSS2 for cell entry, resulting in different neutralization titers depending on TMPRSS2 expression of target cells^32–34^. We were limited by the amount of data and could not stratify by surface receptor expression to test this further. In general, the greatest limitation of aggregating independent datasets is the limited amount of data, the potential of misclassification due to human error, or biased data. In fact, the majority of subjects in the studies were female (Supplementary Fig. S3), calling for attention towards gender-balance in small study cohorts. Studies reported a higher prevalence of severe COVID-19 in males than females, likely due to more B cells and greater antibody base levels and responses in women^35–37^. Further, over a third of all studies came from the US; together with studies from Germany, they made up half of our dataset (Supplementary Fig. S2). Studies from countries from the Global South, and hence people, were massively underrepresented, highlighting research funding and access inequalities. Still, we argue that the trade-off between rapidly available but less-controlled data can be overcome when enough data is used, and has a clear benefit for quick and early pandemic responses. Additionally, an easily accessible database with uniform format and metadata on assay, serum, and localization could greatly contribute to answering questions about SARS-CoV-2 variant specificities for assay standardization, global immunity and public health focus.

Tao et al.^38^. showed the benefit of combining public data to assess BA.1’s and BA.2’s susceptibility to therapeutic monoclonal antibodies. In line with the proposed World Serology Bank by Metcalf et al.^39^, we advocate for a public antigenic database, similar to sequence databases like GenBank and GISAID^40^, where researchers upload virus neutralization and binding data at the time of uploading preprints to an online arXiv. ImmPort^41^ contains antibody neutralization data linked with assay protocols and publications, but, firstly, the variety of data formats and, secondly, the time period from data generation to publication and upload make it less suitable for quick pandemic responses. Alternatively, AI based tools have been employed to create a ‘living evidence map’ of COVID-19^42^, or extract existential risk information from publications^43^. A similar approach could be used to develop and maintain a ‘living antigenic database for SARS-CoV-2’. The development and employment of such technology could easily be applied to other pathogens. Climate change and invasion of humans into animal habitats will only lead to an increase of animal-to-human pathogen transmission; together with the increase in global travel, global pandemics will only become more rather than less likely. We here make the case that an easily accessible public antigenic database, containing neutralization, binding, assay and serum data, would be immensely useful for pandemic preparedness and response.

## Methods

### Data collection

Omicron neutralization data from publicly available preprints (bioRxiv, medRxiv), reports or tweets were collected and metadata regarding serum type (infection history, time since exposure) and assay type (authentic virus, pseudovirus type, cell type) was extracted when available/found in the manuscript. The collected data was reported between 2021/12/08 and 2022/08/14. Mean neutralization fold drops and/or geometric mean neutralization titers were extracted as stated in the respective study’s manuscript. No individual sample data was used, when individual repeat data and the mean was reported we used the mean. Uncertainty of fold change reports was designated by “>” in case of ≥ 50% of samples below the assay’s limit of detection (LOD) of “>>” in case of ≥ 80%. For geometric mean titers (GMTs) the uncertainty was reported with “<” or “<<”, respectively. When no information on the number of detectable samples was given in the manuscript, the uncertainty was assigned by counting from the available data to the extent visual inspection allowed. A full list of all studies considered is shown in Table 1, detailed metadata in the publicly accessible Google Sheets document^3^, also available in the manuscript’s GitHub repository (https://github.com/acorg/netzl_et_al2025/blob/main/data/google_sheet_tables/Netzl%20et%20al.%20-%20Collected%20Omicron%20antigenic%20data.csv).

### Serum group categorization

Serum panels were categorized by their infection or vaccination history into different serum groups. In the “2x Vax” group (n=48) we included double vaccinated individuals, independent of vaccine type, and single dose Johnson & Johnson (J&J) vaccinated individuals, as a single J&J dose is the recommended vaccination regime. The “3x Vax” (n=59) group consisted of triple vaccinated sera, or sera that received a combination of J&J and mRNA vaccines. Individuals with pre-Omicron infection and then vaccination were categorized as “Inf + Vax” (n=24), or vaccination and pre-Omicron breakthrough infection as “Vax + Inf” (n=13). Breakthrough infection with BA.1 was specified as “Vax + BA.1” (n=29) or “Vax + BA.2” (n=6). Convalescent “conv” sera were categorized by the infecting SARS-CoV-2 variant (n(WT)=29, n(Alpha)=8, n(Beta)=6, n(Gamma)=4, n(Delta)=10, n(BA.1)=15, n(BA.2)=4).

### Geometric Mean Titer and fold drop calculation

Geometric Mean Titers (GMT) and mean titer fold drops to a reference antigen were used as stated in each study. In case of thresholded GMTs directly available or extracted from the manuscript, the GMT estimates were set to half the value of the limit of detection (Table 2). Fold drops were calculated by dividing the reference antigen GMT by the variant GMT. Omicron GMTs were obtained by applying the fold drop from wild type to the wild type GMT. Mean fold drops and 95% confidence intervals (95% CI) as reported in Tables 3,4 were obtained by calculating the mean of reported fold drops in each study using Rmisc’s CI() function (v 1.5.1)^44^. Some studies reported fold drops but not titers or variant titers resulting in discrepancies between individual study based mean fold drops and GMT based fold drops. The cumulative mean fold changes were calculated per day.

### Antigenic cartography and antibody landscape slopes since exposure

Antigenic cartography was performed as previously described^19,22,45^. Antigenic maps were constructed in R^46^ version 4.2.2 using the Racmacs^47^ package (v 1.1.35) in 2 dimensions with 1000 optimizations using only convalescent sera. Map diagnostics were performed as previously described and can be found in the supplementary material (Supplementary Fig. S9-S13). Antibody landscapes were constructed as previously described using the ablandscapes^48^ R package (v 1.1.0), ablandscape.fit() function with method=”cone”. This function fits a single cone surface to neutralization titers above an antigenic map. The cone coordinates and apex height is fitted per individual serum, the cone slope is optimized per serum group and quantifies cross-neutralization. A combined slope per serum group reduces overfitting for sera with few, sometimes only two, variant titrations. More variant titrations per serum improve the resolution of the plane across the antigenic map as more geometric information is given to fit the surface. The fraction of titrated variants quantifies this geometric information by dividing the number of measurements by the number of possible measurements (number of map antigens x number of sera). A steep slope indicates poor cross-neutralization. The data was subset to 30 or 90 days since first reported data to construct subset maps and landscapes. To calculate antibody landscape slopes since exposure, sera with information on time since exposure that were titrated against at least two variants were used. The sera were binned into 0.5, 1, 3, 6, 9, and 12 months since exposure. If the time since exposure was more than previous_month + (next_month – previous_month)/2, the serum was assigned to the next_month bin. To quantify the amount of data contributing to each slope, an additional measure of uncertainty, the number of sera n was multiplied with the fraction of available titer measurements, which is calculated by dividing the number of actual titrations by the number of possible measurements. The number of possible measurements is given by n(map variants) x n(serum group sera). To compare data availability across serum groups and times since exposure, the such calculated data amount was scaled by the maximum data amount across serum groups and times since exposure.

## Supplementary Information

Supplementary Information.

## Data Availability

The dataset generated and analysed during the current study is available in the manuscript’s GitHub repository acorg/netzl_et_al2025: Accepted manuscript (v1.0). Zenodo. 10.5281/zenodo.17350682 (https://github.com/acorg/netzl_et_al2025/blob/main/data/google_sheet_tables/Netzl%20et%20al.%20-%20Collected%20Omicron%20antigenic%20data.csv).

## Code availability

All code is publicly available in the manuscript’s GitHub repository acorg/netzl_et_al2025: Accepted manuscript (v1.0). Zenodo. 10.5281/zenodo.17350682 (https://github.com/acorg/netzl_et_al2025.git).
