# Supplementary material for: Combining antigenic data from public sources gives an early indication of the immune escape of emerging virus variants
Source: Sci Rep. 2025 Oct 24;15:37193. doi: 10.1038/s41598-025-19578-3 (PMC12552730; doi:10.1038/s41598-025-19578-3)
Supplement: Supplementary file 1 — Supplementary Information. [file 41598_2025_19578_MOESM1_ESM.pdf]

Supplementary Materials: Combining antigenic data from public sources gives an early indication of the immune escape of emerging virus variants

Antonia Netzl[1], Sina Türeli[1], Eric B. LeGresley[1],
Barbara Mühlemann[2, 3], Samuel H. Wilks[1], Derek J. Smith[1*]

[1*]Centre for Pathogen, Evolution, Department of Zoology, University of Cambridge, Cambridge, United Kingdom.
[2]Institute of Virology, Charité - Universitätsmedizin Berlin, corporate member of Freie Universität Berlin, Humboldt-Universität zu Berlin, and Berlin Institute of Health, Berlin, 10117, Germany.
[3]German Centre for Infection Research (DZIF), partner site Charité, Berlin, 10117, Germany.

*Corresponding author(s). E-mail(s): djs200@cam.ac.uk;

Supplementary Materials

Supplementary Figures

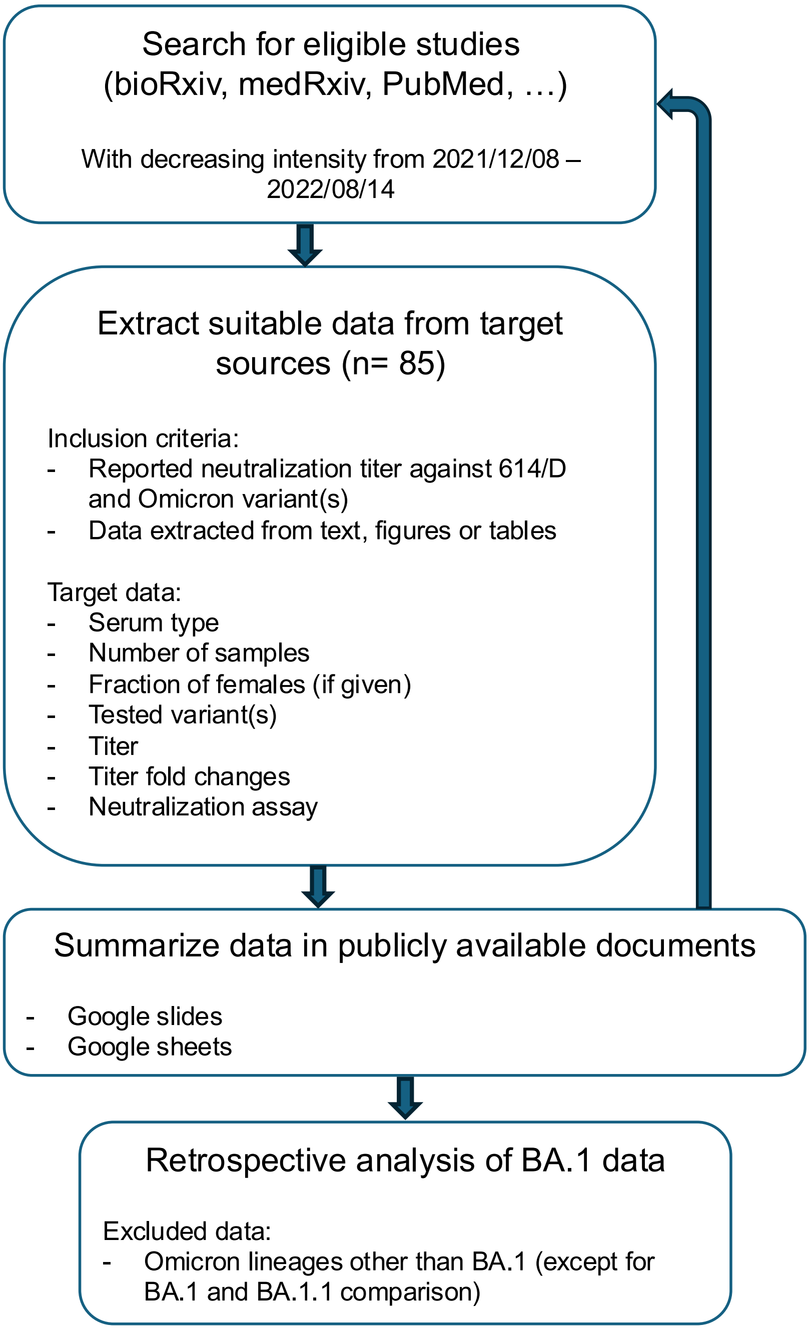

Fig. S1 Flow chart of data extraction and processing. Illustration of the screening and selection process for publicly available data. Data screening, extraction and summary was repeated iteratively throughout the collection period. After the collection period, the retrospective analysis presented in this manuscript was started.

a

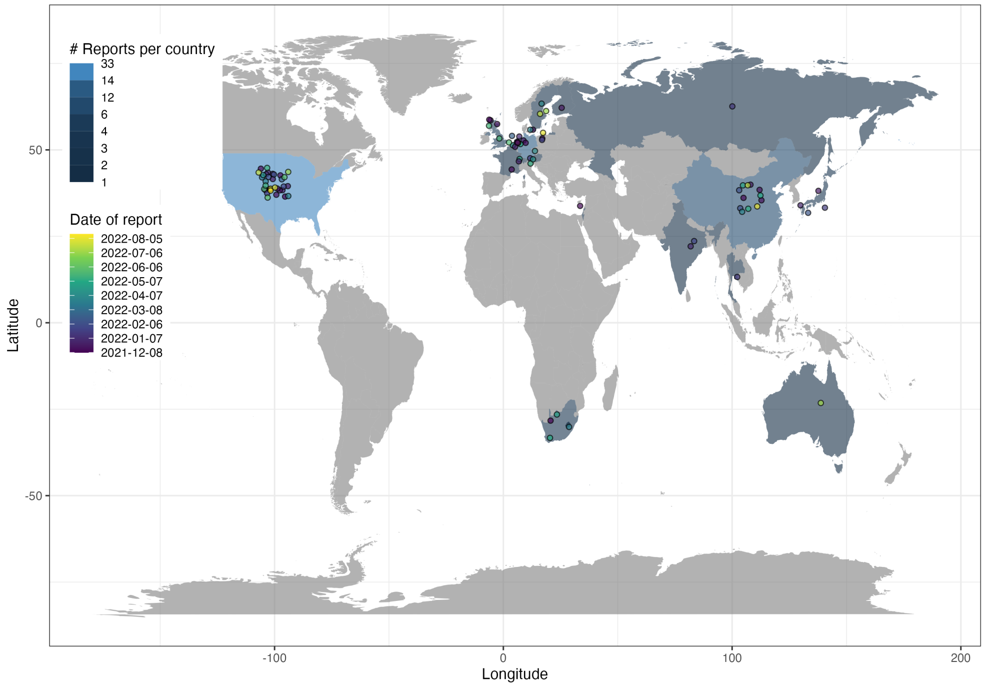

b

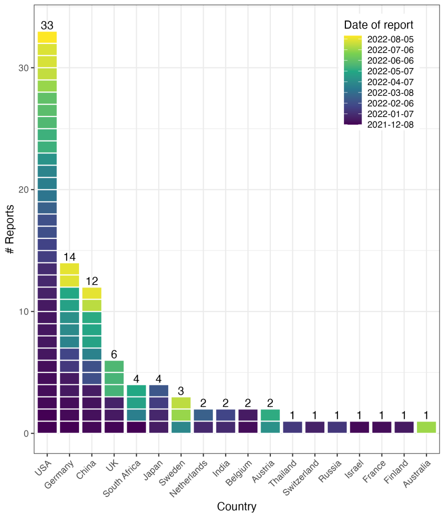

Fig. S2 Studies by location and date. a Individual studies are located in the country of their corresponding authors' institution as colored circles. The color corresponds to the date of online reporting, ranging from blue for early reports to yellow for late reports. The blue country color indicates the number of studies per country, increasing in brightness with increasing number of studies. Grey color indicates that no reports from these countries were used in the current study. **b** Shows the information in **a** in barplot form, ordered by decreasing number of studies per country

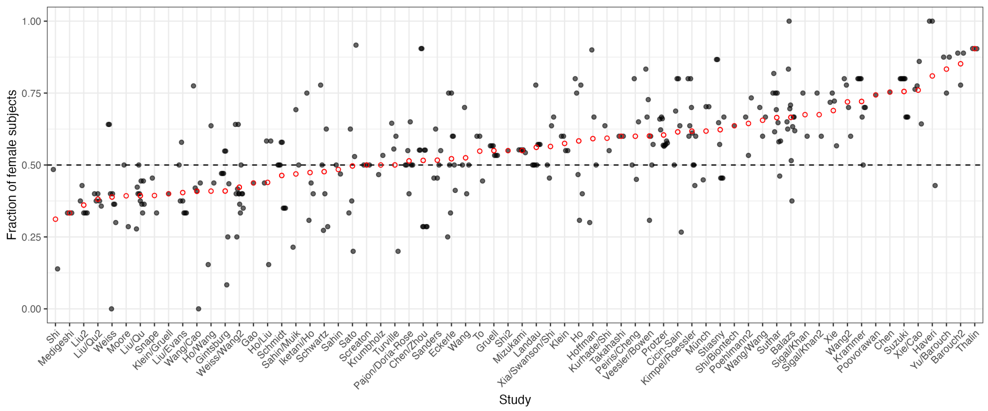

Fig. S3 Fraction of female subjects per study. The fraction of females out of all study subjects was calculated for studies where sex metadata was found. Serum groups per study are shown as black dots with a jitter added on the x-axis to minimize overplotting, the mean fraction of females per study is given by the red, open circle. The dashed line indicates 50%. 29 studies reported data with some or all of the sex metadata missing, 66 studies reported some or all of the sex metadata

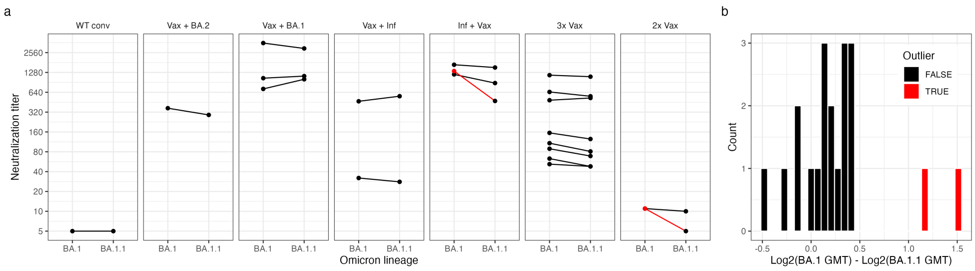

Fig. S4 Neutralization titers of BA.1 and BA.1.1. a) Titers against the two lineages are shown for individual sera in which both lineages were titrated. Measurements from the same sera are connected by lines. The data is stratified by serum group. Outliers based on the log2 difference between BA.1 and BA.1.1 GMTs are shown in red. **b)** A histogram of the log2 titer differences between BA.1 and BA.1.1 in paired samples. A paired t-test showed no significant difference of BA.1 and BA.1.1 GMTs ($p = 0.878$) after removing the two outliers. The outliers were removed as they resulted in an asymmetric distribution of GMT differences that differed significantly from a normal distribution assessed by a Shapiro-Wilk test.

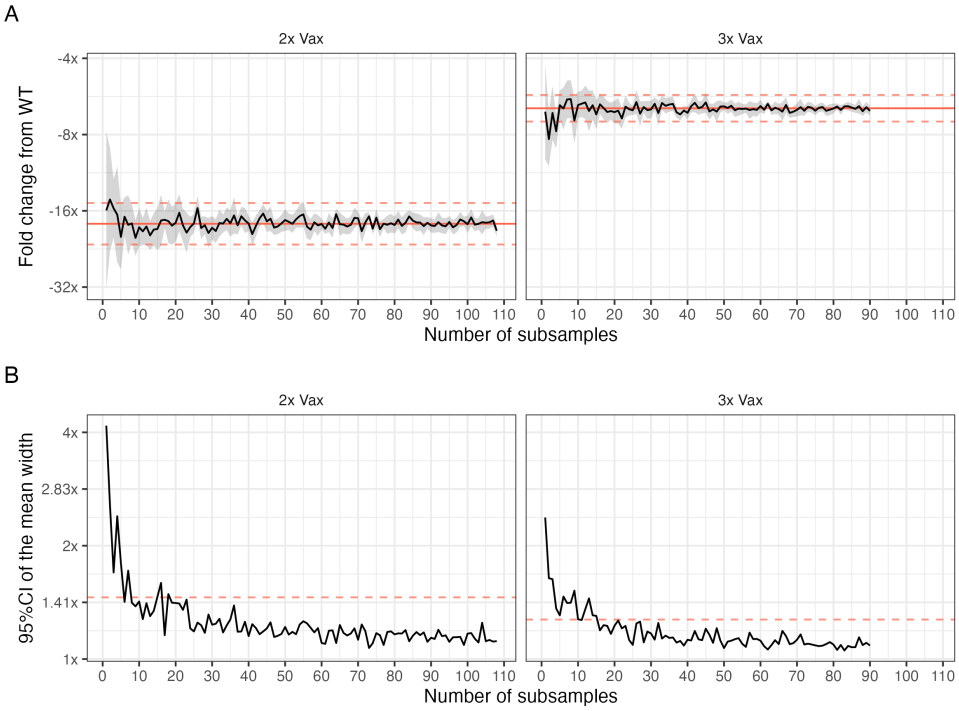

Fig. S5 Bootstrapping the number of studies for BA.1 fold change assessment. A) The titer fold change from WT (614D/G) to BA.1 in the 2x and 3x Vax cohorts was calculated for n=1 to n=total number of studies per cohort by randomly subsampling n studies with replacement. For each n, the random selection was repeated 11 and 9 times in the 2x and 3x Vax cohort respectively, corresponding to 10% of the total number of studies per cohort. The geometric mean and its 95% confidence interval are shown as a solid black line with shaded area. The red solid line shows the mean of all data, the dashed lines indicate the 95%CI. **B)** The range of the such calculated 95%CI of the mean is shown across the number of subsampled studies for the 2x and 3x Vax cohorts, the red dashed lines show the 95% CI range for all data (Upper - Lower 95%CI on the log2 scale, corresponding to Upper/Lower 95%CI on the linear scale)

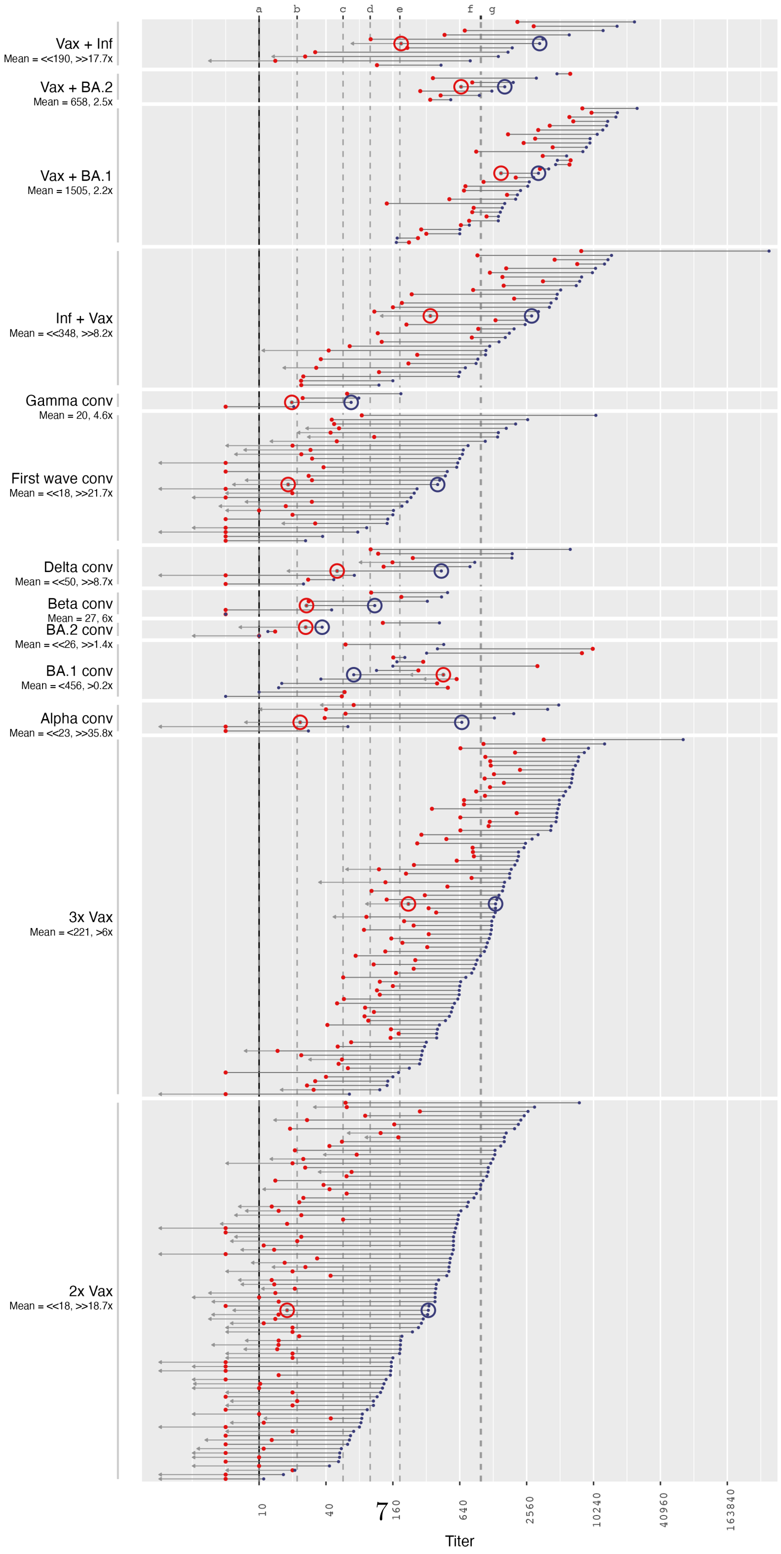

Fig. S6 Omicron BA.1 and WT (614D/G) neutralization titers. GMT neutralization titers as reported by individual studies are shown, ordered by magnitude and stratified by serum group. Red dots indicate Omicron titers and small blue dots indicate WT titers, the big circles indicate GMTs per grouping. Below the serum group label, the BA.1 geometric mean titer (GMT) is given followed by the mean fold change from WT titers. The mean fold change from WT to BA.1 is indicated by the horizontal bar connecting WT and BA.1 titers. Arrows indicate uncertainties in the point estimate due to titers below the limit of detection (LOD)sdf of the assay. A short arrow (>/<) marks groups with more than half of BA.1 titers below the assay's LOD, or conversely reference antigen titers at or lower than the LOD. Long arrows (>>\<<) mark groups with more than approximately 80% of BA.1 titers below the LOD. Dashed lines mark thresholds of protection against symptomatic disease after vaccination with two doses of Moderna (a,d,f,g) [1] or AstraZeneca (b,c,e,f) [2] assessed by pseudovirus neutralization assay (a 78% VE, b 60% VE, c 70% VE, d 91% VE, e 80% VE, f 90% VE, g 96% VE)

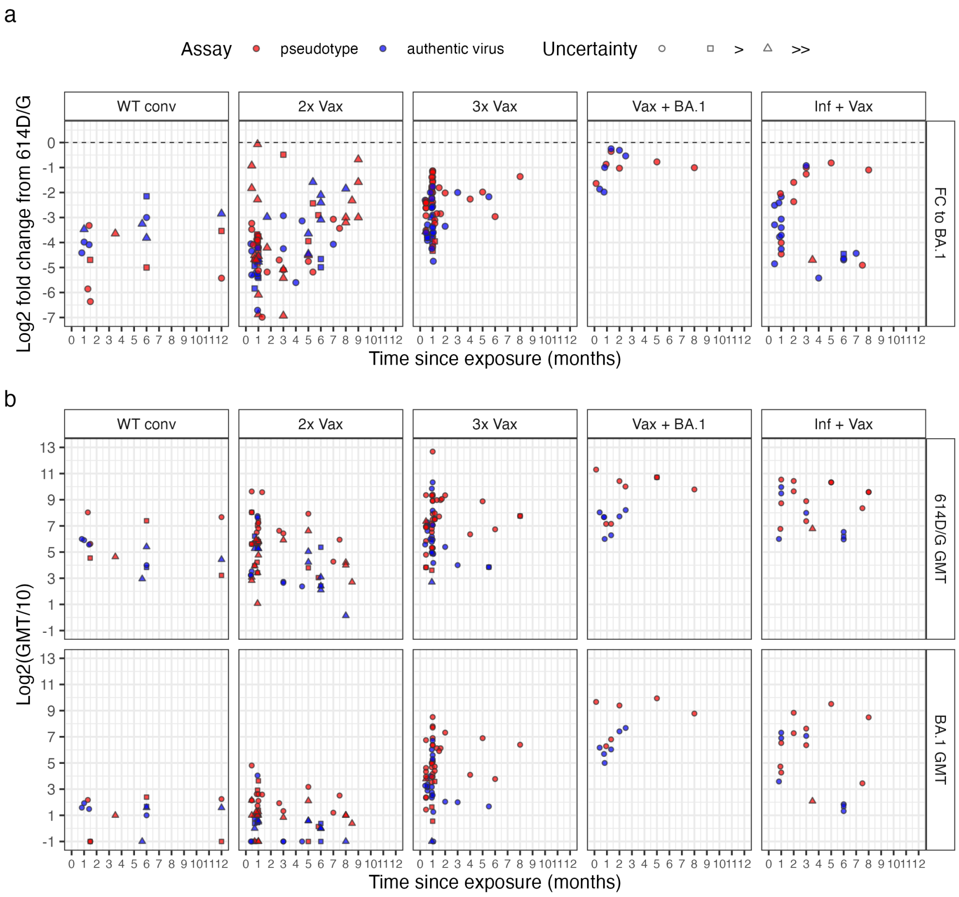

Fig. S7 Individual studies by time since last exposure. a) 614D/G (WT) to BA.1 log2 fold changes by time since last exposure and **b)** geometric mean titers (GMTs) for 614D/G (WT) and BA.1 by time since last exposure in the individual studies which listed time since exposure information. The data is stratified by exposure history. Shapes indicate the level of uncertainty due to the fraction of samples below the level of detection (LOD) for each mean value shown (> : more than or equal to 50% of BA.1 or 614D/G titers below LOD; >> : more than or equal to 80% of BA.1 or 614D/G below LOD. Assessed by visual inspection if no sample number was given). Data that was generated with a pseudotyped virus is shown in red, data that was generated in an authentic virus assay in blue.

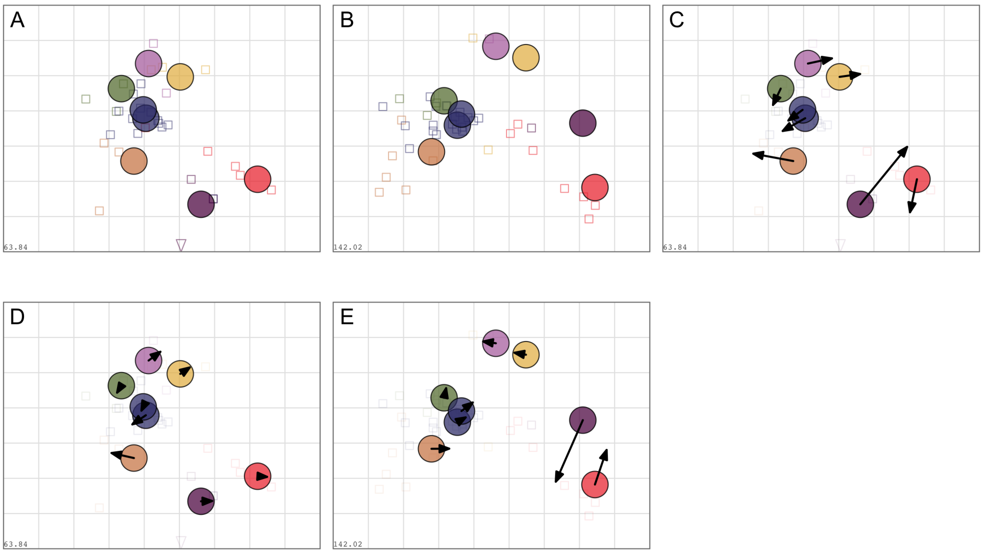

Fig. S8 Antigenic maps from authentic virus and pseudovirus data. Antigenic maps were constructed as described in the methods section. **A)** A map was constructed using only data from authentic virus neutralization assays and **B)** using only data from pseudovirus neutralization assays. **C)** Shows a comparison of the two maps with arrows pointing from the variant position in map A to the variant position in map B. **D)** Shows the same comparison but with arrows pointing from A to the full data map shown in Fig. 3b. **E)** Shows a comparison of map B with arrows pointing to the full data map shown in Fig. 3b

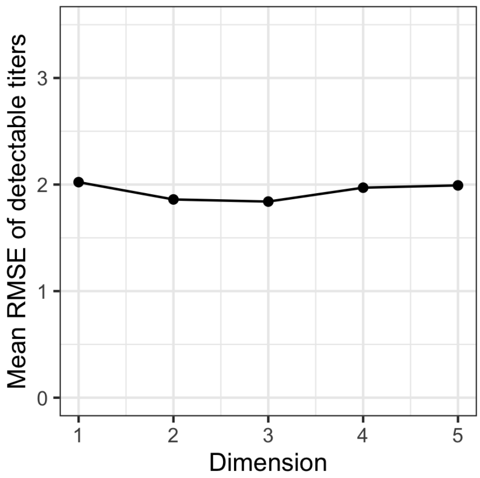

Fig. S9 Dimensionality Test. Root Mean Squared Error (RMSE) between map and measured titers for detectable titers in 1 to 5 dimensions. Per dimension, 100 map replicates were constructed from 90% of measured titers with 1000 optimizations per replicate. The titers of the remaining 10% were predicted in each run and the RMSE calculated by comparing the predicted to the measured titers on the $\log_2$ scale

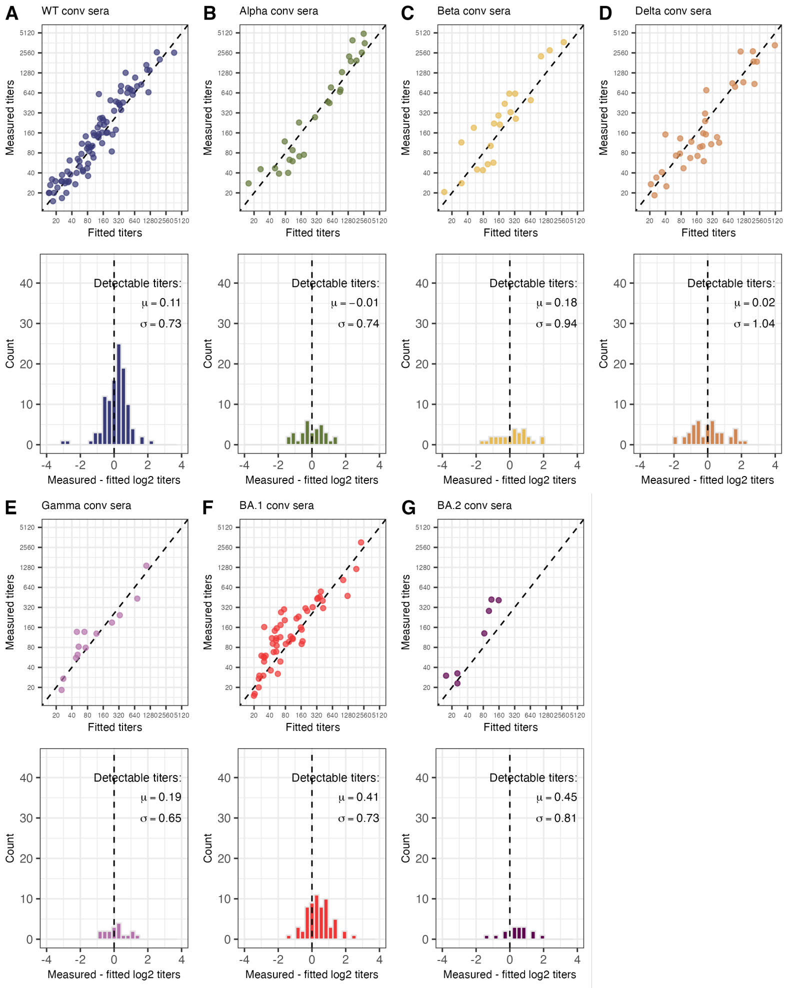

Fig. S10 Goodness of map fit per serum group. The top panels show the correlation of detectable measured and fitted titers in the 2D map (Fig. 3b). Map distances were converted into $\log_2$ titers by subtracting the Euclidean distance for each serum-antigen pair from the maximum $\log_2$ titer of the specific serum. The bottom panels show the residuals of measured against fitted titers on the $\log_2$ scale, light grey marks pairs with the measured titer below the assay detection threshold. The mean and mean-centered standard deviation of differences between fitted and detectable measured titers are given in the legend of each bottom row panel. This was done for the serum groups used to construct the map: Infected with **A** WT (614D/G), **B** Alpha, **C** Beta, **D** Delta, **E** Gamma, **F** BA.1. and **G** BA.2

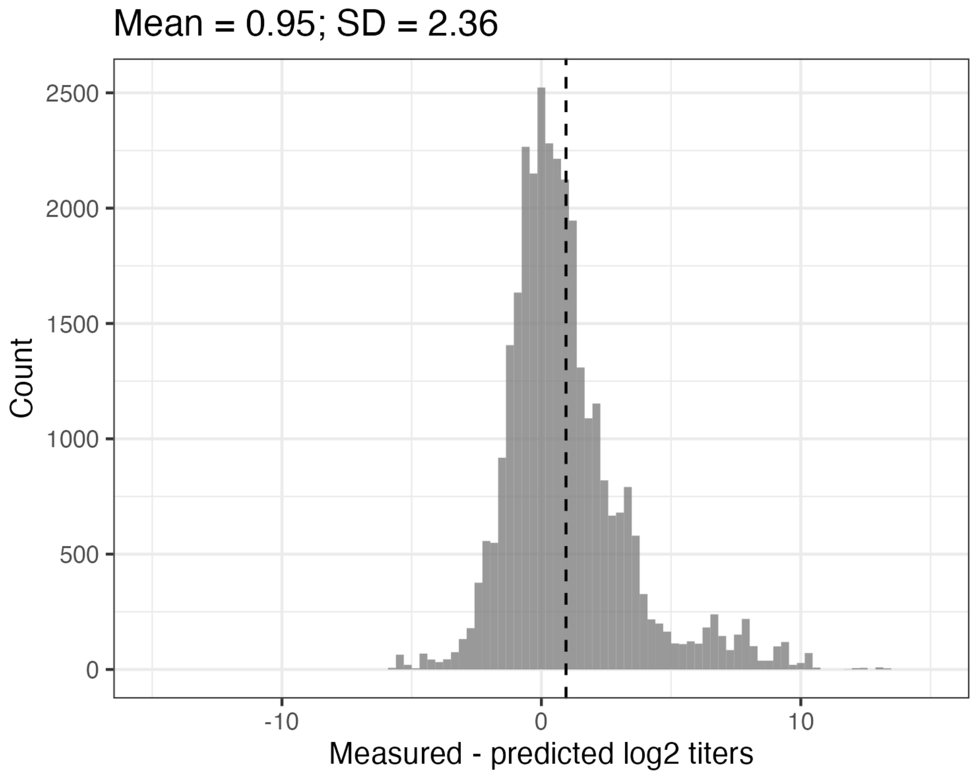

Fig. S11 Map cross-validation residual titers. 1000 repeats with 1000 optimization runs each were performed with only 90% of measured titers used for map construction by artificially masking 10% of measurements. The missing $\log_2$ titers were predicted by subtracting the Euclidean map distance for each serum-antigen pair from the maximum $\log_2$ titer of the specific serum. The difference between predicted and detectable measured titers on the $\log_2$ scale was calculated, the mean is indicated by the dashed line. The mean and mean-centered standard deviation are given in the title

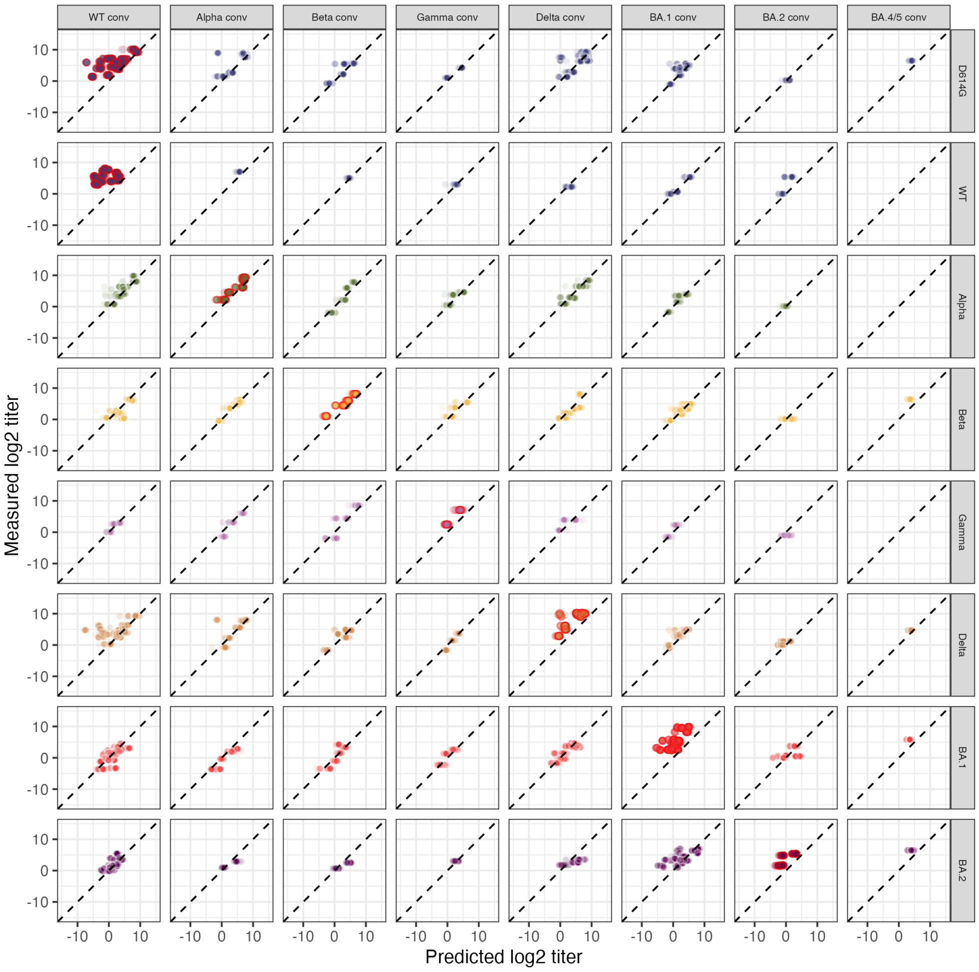

Fig. S12 Predicted vs. measured titers. 1000 repeats with 1000 optimization runs each were performed with only 90% of measured titers used for map construction by artificially masking 10% of measurements. The missing $\log_2$ titers/10 were predicted by subtracting the Euclidean map distance for each serum-antigen pair from the maximum $\log_2$ titer/10 of the specific serum. Measured over predicted $\log_2$ titers/10 are shown per serum group and antigen variant. Virus variants are shown as rows, serum groups as columns. The points are coloured by variant and a red outline is added for the homologous serum-antigen pairs

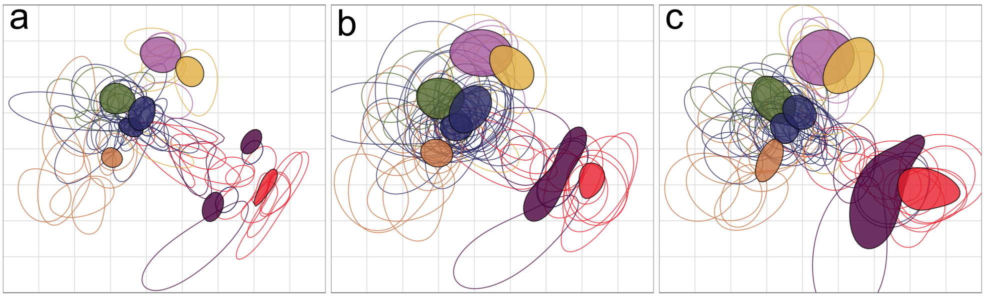

Fig. S13 Positional resolution by bootstrapping. 500 bootstrap repeats were performed with 1000 optimizations per repeat and options as listed in the Methods section. In each repeat, **a)** different weights are assigned to sera and antigen reactivity of the titer table. The weights are drawn randomly from a Dirichlet distribution. **b)** Sera and antigens are randomly resampled with replacement, and **c)** normally distributed noise with sd = 0.7 is added to antigens and individual titer measurements. The colored regions mark 68% (one standard deviation) of the positional variation for each variant (filled shapes) and sera (open shapes). The colors correspond to the colors used in Fig. 3

Supplementary Tables

Table S1: **Comparison of preprint data and published data.** For eight randomly selected studies, titers that were given in the preprint were compared to titers reported in the peer-reviewed publication. The studies are named in accordance to the reporting in this manuscript. The sources reference both preprint and publication, in two cases the initially extracted data was already peer-reviewed and published. For each serum, the number of samples is given for preprint and publication, followed by the titers in preprint and publication. Discrepancies between preprint and publication are highlighted in bold.

Study	Preprint Source, Publication Source	Serum	Number of sera Preprint	Number of sera Publication	Variant	Titers Preprint	Titers Publication
Chen	[3], [4]	2*CoronaVac + CoronaVac (1m post 3rd dose)	292	292	WT	294.85	294.85
					BA.1	14.66	14.66
Gao	[5], [6]	2*CoronaVac + CoronaVac	16	16	WT	284	284
					BA.1	56	56
		WT convalescent	16	16	WT	193	193
					BA.1	11.1	11.1
Klein	[7], [8]	2*Pfizer	30	30	WT	561	561
					BA.1	11	11
					BA.1.1	8	8
					BA.2	15	15
					BA.2.12.1	17	17
					BA.4/5	11	11

Continued on next page

Table S1: **Comparison of preprint data and published data.** For eight randomly selected studies, titers that were given in the preprint were compared to titers reported in the peer-reviewed publication. The studies are named in accordance to the reporting in this manuscript. The sources reference both preprint and publication, in two cases the initially extracted data was already peer-reviewed and published. For each serum, the number of samples is given for preprint and publication, followed by the titers in preprint and publication. Discrepancies between preprint and publication are highlighted in bold. (Continued)

Study	Preprint Source, Publication Source	Serum	Number of sera Preprint	Number of sera Publication	Variant	Titers Preprint	Titers Publication
		3*Pfizer	30	30	WT	4773	4773
					BA.1	648	648
					BA.1.1	557	557
					BA.2	582	582
					BA.2.12.1	465	465
					BA.4/5	312	312
		WT conv	20	20	WT	264	264
					BA.1	6	6
					BA.1.1	5	5
					BA.2	11	11
					BA.2.12.1	12	12
					BA.4/5	11	11
		WT conv + Pfizer	20	20	WT	10671	10671
					BA.1	1674	1674
					BA.1.1	1521	1521
					BA.2	2103	2103
					BA.2.12.1	1946	1946
					BA.4/5	1456	1456

Continued on next page

Table S1: **Comparison of preprint data and published data.** For eight randomly selected studies, titers that were given in the preprint were compared to titers reported in the peer-reviewed publication. The studies are named in accordance to the reporting in this manuscript. The sources reference both preprint and publication, in two cases the initially extracted data was already peer-reviewed and published. For each serum, the number of samples is given for preprint and publication, followed by the titers in preprint and publication. Discrepancies between preprint and publication are highlighted in bold. (Continued)

Study	Preprint Source, Publication Source	Serum	Number of sera Preprint	Number of sera Publication	Variant	Titers Preprint	Titers Publication
Kurhade/Shi	[9], [10]	3*Pfizer (1m post 3rd dose)	22	22	WT	1335	1335
					BA.1	393	393
					BA.2	298	298
					BA.2.12.1	315	315
					BA.3	216	216
					BA.4/5	103	103
		BA.1 conv	20	20	WT	15	15
					BA.1	430	430
					BA.2	110	110
					BA.2.12.1	109	109
					BA.3	102	102
					BA.4/5	25	25
Screaton	[11] (already published)	2*AZ (28d post 2nd dose)	41	41	WT	390	390
					BA.1	21	21

Continued on next page

Table S1: **Comparison of preprint data and published data.** For eight randomly selected studies, titers that were given in the preprint were compared to titers reported in the peer-reviewed publication. The studies are named in accordance to the reporting in this manuscript. The sources reference both preprint and publication, in two cases the initially extracted data was already peer-reviewed and published. For each serum, the number of samples is given for preprint and publication, followed by the titers in preprint and publication. Discrepancies between preprint and publication are highlighted in bold. (Continued)

**Study**	**Preprint Source, Publication Source**	**Serum**	**Number of sera Preprint**	**Number of sera Publication**	**Variant**	**Titers Preprint**	**Titers Publication**
		2*AZ + AZ (28d post 3rd dose)	41	41	WT	726	726
					BA.1	57	57
		2*Pfizer (28d post 2nd dose)	20	20	WT	1993	1993
					BA.1	19	19
		2*Pfizer (6m post 2nd dose)	20	20	WT	413	413
					BA.1	13	13
		2*Pfizer + Pfizer (28d post 3rd dose)	20	20	WT	9219	9219
					BA.1	649	649

Continued on next page

Table S1: **Comparison of preprint data and published data.** For eight randomly selected studies, titers that were given in the preprint were compared to titers reported in the peer-reviewed publication. The studies are named in accordance to the reporting in this manuscript. The sources reference both preprint and publication, in two cases the initially extracted data was already peer-reviewed and published. For each serum, the number of samples is given for preprint and publication, followed by the titers in preprint and publication. Discrepancies between preprint and publication are highlighted in bold. (Continued)

**Study**	**Preprint Source, Publication Source**	**Serum**	**Number of sera Preprint**	**Number of sera Publication**	**Variant**	**Titers Preprint**	**Titers Publication**
		Alpha convalescent (18d post inf)	18	18	WT	1313	1313
					BA.1	39	39
		Beta convalescent (61d post inf)	14	14	WT	327	327
					BA.1	28	28
		Delta convalescent (38d post inf)	19	19	WT	47	47
					BA.1	27	27
		Delta inf + vacc/ vacc + inf	17	17	WT	1899	1899

Continued on next page

Table S1: **Comparison of preprint data and published data.** For eight randomly selected studies, titers that were given in the preprint were compared to titers reported in the peer-reviewed publication. The studies are named in accordance to the reporting in this manuscript. The sources reference both preprint and publication, in two cases the initially extracted data was already peer-reviewed and published. For each serum, the number of samples is given for preprint and publication, followed by the titers in preprint and publication. Discrepancies between preprint and publication are highlighted in bold. (Continued)

Study	Preprint Source, Publication Source	Serum	Number of sera Preprint	Number of sera Publication	Variant	Titers Preprint	Titers Publication
					BA.1	215	215
		Gamma convalescent (63d post inf)	16	16	WT	79	79
					BA.1	25	25
		WT convalasecent (42d post inf)	32	32	WT	475	475
					BA.1	28	28
Stiasny	[12], [13]	3*mRNA (3-4w post 3rd dose)	15	15	WT	640	640
					BA.1	160	160
					BA.2	160	160
		3*mRNA (3m post 3rd dose)	15	15	WT	160	160

Continued on next page

Table S1: **Comparison of preprint data and published data.** For eight randomly selected studies, titers that were given in the preprint were compared to titers reported in the peer-reviewed publication. The studies are named in accordance to the reporting in this manuscript. The sources reference both preprint and publication, in two cases the initially extracted data was already peer-reviewed and published. For each serum, the number of samples is given for preprint and publication, followed by the titers in preprint and publication. Discrepancies between preprint and publication are highlighted in bold. (Continued)

Study	Preprint Source, Publication Source	Serum	Number of sera Preprint	Number of sera Publication	Variant	Titers Preprint	Titers Publication
					BA.1	40	40
					BA.2	60	60
		BA.1 Conv (3-4w post inf)	**18**	**22**	WT	10	10
					BA.1	60	60
					**BA.2**	**20**	**17.5**
		BA.2 Conv (3-4w post inf)	**7**	**21**	WT	10	10
					BA.1	10	10
					**BA.2**	**30**	**20**
		Omicron Break-through (3-4w post inf)	**11**	**43**	**WT**	**640**	**960**
					BA.1	320	320
					**BA.2**	**480**	**640**

Continued on next page

Table S1: **Comparison of preprint data and published data.** For eight randomly selected studies, titers that were given in the preprint were compared to titers reported in the peer-reviewed publication. The studies are named in accordance to the reporting in this manuscript. The sources reference both preprint and publication, in two cases the initially extracted data was already peer-reviewed and published. For each serum, the number of samples is given for preprint and publication, followed by the titers in preprint and publication. Discrepancies between preprint and publication are highlighted in bold. (Continued)

**Study**	**Preprint Source, Publication Source**	**Serum**	**Number of sera Preprint**	**Number of sera Publication**	**Variant**	**Titers Preprint**	**Titers Publication**
		WT Conv (3-4w post inf)	11	11	WT	640	640
					BA.1	30	30
					BA.2	40	40
		WT Conv (6m post inf)	11	11	WT	160	160
					BA.1	20	20
					BA.2	30	30
		WT Inf + 3*mRNA (3-4w post)	9	9	WT	640	640
					BA.1	120	120
					BA.2	240	240
To	[14] (already published)	BA.2 conv	10	10	BA.1	16.3	16.3

Continued on next page

Table S1: **Comparison of preprint data and published data.** For eight randomly selected studies, titers that were given in the preprint were compared to titers reported in the peer-reviewed publication. The studies are named in accordance to the reporting in this manuscript. The sources reference both preprint and publication, in two cases the initially extracted data was already peer-reviewed and published. For each serum, the number of samples is given for preprint and publication, followed by the titers in preprint and publication. Discrepancies between preprint and publication are highlighted in bold. (Continued)

**Study**	**Preprint Source, Publication Source**	**Serum**	**Number of sera Preprint**	**Number of sera Publication**	**Variant**	**Titers Preprint**	**Titers Publication**
					BA.2	32.5	32.5
		Non-omicron + Pfizer	15	15	BA.1	12.6	12.6
					BA.2	29.4	29.4
		Non-omicron conv	9	9	BA.1	211	211
					BA.2	422	422
Wang/Cao	[15], [16]	3 * CoronaVac	40	40	D614G	652	652
					BA.1	122	122
					BA.2	130	130
					BA.2.12.1	105	105
					BA.4/5	72	72
					BA.2.38	124	124
					BA.2.75	90	90
					BA.2.76	93	93
		3 * CoronaVac + BA.1	50	50	D614G	1545	1545

Continued on next page

Table S1: **Comparison of preprint data and published data.** For eight randomly selected studies, titers that were given in the preprint were compared to titers reported in the peer-reviewed publication. The studies are named in accordance to the reporting in this manuscript. The sources reference both preprint and publication, in two cases the initially extracted data was already peer-reviewed and published. For each serum, the number of samples is given for preprint and publication, followed by the titers in preprint and publication. Discrepancies between preprint and publication are highlighted in bold. (Continued)

Study	Preprint Source, Publication Source	Serum	Number of sera Preprint	Number of sera Publication	Variant	Titers Preprint	Titers Publication
					BA.1	852	852
					BA.2	452	452
					BA.2.12.1	244	244
					BA.4/5	107	107
					BA.2.38	443	443
					BA.2.75	197	197
					BA.2.76	205	205
		3 * CoronaVac + BA.2	39	39	D614G	1245	1245
					BA.1	282	282
					BA.2	696	696
					BA.2.12.1	290	290
					BA.4/5	175	175
					BA.2.38	613	613
					BA.2.75	217	217
					BA.2.76	227	227
		3 * CoronaVac + BA.5	8	8	D614G	1401	1401
					BA.1	161	161

Continued on next page

Table S1: **Comparison of preprint data and published data.** For eight randomly selected studies, titers that were given in the preprint were compared to titers reported in the peer-reviewed publication. The studies are named in accordance to the reporting in this manuscript. The sources reference both preprint and publication, in two cases the initially extracted data was already peer-reviewed and published. For each serum, the number of samples is given for preprint and publication, followed by the titers in preprint and publication. Discrepancies between preprint and publication are highlighted in bold. (Continued)

**Study**	**Preprint Source, Publication Source**	**Serum**	**Number of sera Preprint**	**Number of sera Publication**	**Variant**	**Titers Preprint**	**Titers Publication**
					BA.2	811	811
					BA.2.12.1	691	691
					BA.4/5	481	481
					BA.2.38	755	755
					BA.2.75	115	115
					BA.2.76	221	221
		3 * CoronaVac + Delta	16	16	D614G	434	434
					BA.1	115	115
					BA.2	140	140
					BA.2.12.1	125	125
					BA.4/5	90	90
					BA.2.38	135	135
					BA.2.75	67	67
					BA.2.76	75	75

Table S2: **Geometric mean titer of BA.1 and BA.1.1.** GMTs from individual sera that titrated both BA.1 and BA.1.1 are shown with 95% confidence intervals

Serum group	BA.1	BA.1.1	WT
2x Vax	11 (11; 11)<br>n=2	5 (0; 578)<br>n=2	176 (0; 449161905)<br>n=2
3x Vax	191 (72; 506)<br>n=8	164 (58; 463)<br>n=8	1022 (370; 2827)<br>n=8
Inf + Vax	1390 (908; 2129)<br>n=3	859 (201; 3672)<br>n=3	6481 (871; 48257)<br>n=3
Vax + Inf	122 (0; 3045502164)<br>n=2	125 (0; 22510158116)<br>n=2	3281 (1; 10390008)<br>n=2
Vax + BA.1	1392 (174; 11161)<br>n=3	1498 (344; 6528)<br>n=3	3462 (1115; 10751)<br>n=3
Vax + BA.2	367<br>n=1	290<br>n=1	3130<br>n=1
WT conv	5<br>n=1	5<br>n=1	264<br>n=1

Table S3: **Assay-based statistical comparison of live virus vs pseudovirus fold changes from WT/D614G to BA.1**. A t-test was performed to compare pseudovirus (PV) and live virus (LV) assessed fold changes in different serum groups. Normality was checked with a Shapiro-Wilk test and these serum groups were found to not differ significantly from a normal distribution. Results are ordered by increasing p-value. A statistic above 0 indicates higher values in PV than LV. The effect size was calculated with Cohen's d for unequal variances and Hedge's correction due to small sample sizes. All tests were performed with the rstatix package [17]

Serum group	n(PV)	n(LV)	statistic	95%CI (lower;upper)	df	p	Significance	Effect size	Magnitude
3x Vax	61	28	-4.39	-1.11;-0.42	51.4	0.00	****	-1.00	large
WT conv	19	14	2.99	0.35;1.83	30.9	0.01	**	1.01	large

Continued on next page

Table S3: **Assay-based statistical comparison of live virus vs pseudovirus fold changes from WT/D614G to BA.1**. A t-test was performed to compare pseudovirus (PV) and live virus (LV) assessed fold changes in different serum groups. Normality was checked with a Shapiro-Wilk test and these serum groups were found to not differ significantly from a normal distribution. Results are ordered by increasing p-value. A statistic above 0 indicates higher values in PV than LV. The effect size was calculated with Cohen's d for unequal variances and Hedge's correction due to small sample sizes. All tests were performed with the rstatix package [17] (Continued)

**Serum group**	**n(PV)**	**n(LV)**	**statistic**	**95%CI (lower;upper)**	**df**	**p**	**Significance**	**Effect size**	**Magnitude**
2x Vax	69	39	-1.58	-0.98;0.11	88.1	0.12	ns	-0.31	small
Beta conv	3	3	-0.97	-3.51;2.02	2.5	0.42	ns	-0.64	moderate
Vax + Inf	10	5	-0.84	-2.31;1.1	6.9	0.43	ns	-0.45	small
Vax + BA.1	25	7	-0.59	-1.4;0.83	8.2	0.57	ns	-0.26	small
Delta conv	7	3	0.47	-2.14;3.12	5.2	0.66	ns	0.28	small

Table S4: **Assay-based statistical comparison of live virus vs pseudovirus fold changes from WT/D614G to BA.1.** A Wilcoxon-test was performed to compare pseudovirus (PV) and live virus (LV) assessed fold changes in different serum groups. Normality was checked with a Shapiro-Wilk test and these serum groups were found to differ significantly from a normal distribution. Results are ordered by increasing p-value. A statistic above 0 indicates higher values in PV than LV. The effect size was calculated with Wilcoxon effect size. All tests were performed with the rstatix package [17]

**Serum group**	**n(PV)**	**n(LV)**	**statistic**	**95%CI (lower;upper)**	**p**	**Significance**	**Effect size**	**Magnitude**
Inf + Vax	17	23	138.5	-2.03;0.2	0.12	ns	0.25	small
BA.1 conv	6	5	12.0	-6.99;2.9	0.65	ns	0.17	small
Alpha conv	5	3	9.0	-1.74;3.65	0.79	ns	0.16	small

Table S5: **Assay-based statistical comparison of live virus vs pseudo virus variant GMTs.** A t-test was performed to compare pseudovirus (PV) and live virus (LV) assessed Geometric Mean Titers (GMTs) in different serum groups. Normality was checked with a Shapiro-Wilk test and these serum groups were found to not differ significantly from a normal distribution. Results are ordered by increasing p-value. A statistic above 0 indicates higher values in PV than LV. The effect size was calculated with Cohen's d for unequal variances and Hedge's correction due to small sample sizes. All tests were performed with the rstatix package [17]

Variant	Serum group	n(PV)	n(LV)	statistic	95%CI (lower;upper)	df	p	Significance	Effect size	Magnitude
BA.1	3x Vax	58	23	4.48	1.12;2.95	38.8	0.0001	****	1.10	large
WT	Delta conv	6	3	8.56	3.85;6.96	5.8	0.0002	***	5.05	large
BA.1	Vax + BA.1	24	12	3.74	0.76;2.56	32.2	0.0007	***	1.19	large
BA.1	Inf + Vax	17	15	3.65	1.27;4.52	29.3	0.0010	**	1.26	large
WT	3x Vax	62	24	3.24	0.52;2.22	41.8	0.0024	**	0.77	moderate
WT	WT conv	20	12	3.24	0.75;3.37	25.1	0.0033	**	1.14	large
Delta	WT conv	10	8	2.59	0.42;4.2	15.3	0.0201	*	1.14	large
WT	2x Vax	61	32	2.35	0.15;1.83	58.7	0.0220	*	0.52	moderate
Delta	Delta conv	6	3	5.27	1.2;9.06	2.1	0.0293	*	3.76	large
Beta	3x Vax	16	11	2.26	0.13;3.04	20.9	0.0346	*	0.86	large
Alpha	WT conv	5	3	2.62	0.21;7.6	5.7	0.0415	*	1.59	large
Beta	Inf + Vax	3	6	3.18	-0.96;7.3	2.1	0.0819	ns	2.29	large

Continued on next page

Table S5: **Assay-based statistical comparison of live virus vs pseudo virus variant GMTs.** A t-test was performed to compare pseudovirus (PV) and live virus (LV) assessed Geometric Mean Titers (GMTs) in different serum groups. Normality was checked with a Shapiro-Wilk test and these serum groups were found to not differ significantly from a normal distribution. Results are ordered by increasing p-value. A statistic above 0 indicates higher values in PV than LV. The effect size was calculated with Cohen's d for unequal variances and Hedge's correction due to small sample sizes. All tests were performed with the rstatix package [17] (Continued)

Variant	Serum group	n(PV)	n(LV)	statistic	95%CI (lower;upper)	df	p	Significance	Effect size	Magnitude
Delta	Alpha conv	3	3	2.92	-2.55;13.64	2.0	0.0989	ns	1.91	large
Beta	Delta conv	3	3	2.54	-1.7;9.85	2.5	0.1020	ns	1.66	large
WT	Alpha conv	3	3	2.69	-2.12;11.47	2.2	0.1030	ns	1.76	large
Delta	Vax + Inf	5	6	1.83	-0.47;4.21	8.4	0.1030	ns	0.99	large
WT	BA.1 conv	7	5	1.85	-0.93;5.66	5.0	0.1240	ns	1.05	large
WT	Vax + Inf	7	4	1.60	-0.68;4.01	9.0	0.1430	ns	0.85	large
BA.1	Vax + Inf	6	6	1.56	-0.92;5.09	9.1	0.1520	ns	0.83	large
Beta	WT conv	6	3	1.57	-0.91;4.42	6.7	0.1620	ns	0.85	large
Delta	2x Vax	25	19	1.40	-0.36;1.93	34.1	0.1700	ns	0.42	small
Delta	BA.1 conv	5	4	1.31	-2.23;6	3.7	0.2650	ns	0.81	large
Beta	2x Vax	24	17	0.76	-0.67;1.45	29.2	0.4540	ns	0.24	small
Beta	BA.1 conv	4	3	0.81	-3.89;6.49	2.9	0.4780	ns	0.54	moderate
Alpha	2x Vax	4	7	-0.69	-4;2.29	5.1	0.5180	ns	-0.41	small

Continued on next page

Table S5: **Assay-based statistical comparison of live virus vs pseudo virus variant GMTs.** A t-test was performed to compare pseudovirus (PV) and live virus (LV) assessed Geometric Mean Titers (GMTs) in different serum groups. Normality was checked with a Shapiro-Wilk test and these serum groups were found to not differ significantly from a normal distribution. Results are ordered by increasing p-value. A statistic above 0 indicates higher values in PV than LV. The effect size was calculated with Cohen's d for unequal variances and Hedge's correction due to small sample sizes. All tests were performed with the rstatix package [17] (Continued)

Variant	Serum group	n(PV)	n(LV)	statistic	95%CI (lower;upper)	df	p	Significance	Effect size	Magnitude
Delta	3x Vax	18	13	0.65	-1.02;1.97	25.1	0.5190	ns	0.23	small
Delta	Vax + BA.1	9	7	-0.24	-2.3;1.84	10.2	0.8120	ns	-0.11	negligible

Table S6: **Assay-based statistical comparison of live virus vs pseudovirus variant GMTs.** A Wilcoxon-test was performed to compare pseudovirus (PV) and live virus (LV) assessed Geometric Mean Titers (GMTs) in different serum groups. Normality was checked with a Shapiro-Wilk test and these serum groups were found to differ significantly from a normal distribution. Results are ordered by increasing p-value. A statistic above 0 indicates higher values in PV than LV. The effect size was calculated with Wilcoxon effect size. All tests were performed with the rstatix package [17]

Variant	Serum group	n(PV)	n(LV)	statistic	95%CI (lower;upper)	p	Significance	Effect size	Magnitude
BA.1	2x Vax	55	26	1095.5	0.5;1.56	0.0001	***	0.43	moderate
WT	Inf + Vax	17	15	200.0	0.58;3.1	0.0053	**	0.48	moderate
BA.1	BA.1 conv	6	8	42.0	0.14;5.74	0.0200	*	0.62	large
BA.1	Delta conv	5	3	15.0	1.87;5.6	0.0358	*	0.80	large
BA.1	Alpha conv	3	3	9.0	0.04;3.83	0.0765	ns	0.81	large

Continued on next page

Table S6: **Assay-based statistical comparison of live virus vs pseudovirus variant GMTs.** A Wilcoxon-test was performed to compare pseudovirus (PV) and live virus (LV) assessed Geometric Mean Titers (GMTs) in different serum groups. Normality was checked with a Shapiro-Wilk test and these serum groups were found to differ significantly from a normal distribution. Results are ordered by increasing p-value. A statistic above 0 indicates higher values in PV than LV. The effect size was calculated with Wilcoxon effect size. All tests were performed with the rstatix package [17] (Continued)

**Variant**	**Serum group**	**n(PV)**	**n(LV)**	**statistic**	**95%CI (lower;upper)**	**p**	**Significance**	**Effect size**	**Magnitude**
Alpha	Delta conv	3	3	9.0	3.52;7.5	0.1000	ns	0.80	large
WT	Vax + BA.1	27	7	133.0	-0.28;2.66	0.1070	ns	0.28	small
Beta	Vax + BA.1	7	3	16.0	-2.8;4.17	0.2670	ns	0.40	moderate
Delta	Inf + Vax	7	9	36.0	-1.42;1.6	0.6810	ns	0.12	small
BA.1	WT conv	15	13	102.5	-0.88;1.49	0.8350	ns	0.04	small

References

[1] Gilbert, P. B. *et al.* Immune Correlates Analysis of the mRNA-1273 COVID-19 Vaccine Efficacy Trial (2021). URL https://www.medrxiv.org/content/10.1101/2021.08.09.21261290v4. Pages: 2021.08.09.21261290.

[2] Feng, S. *et al.* Correlates of protection against symptomatic and asymptomatic SARS-CoV-2 infection. *Nature Medicine* **27**, 2032–2040 (2021).

[3] Yu, X. *et al.* Reduced sensitivity of SARS-CoV-2 Omicron variant to booster-enhanced neutralization (2021). URL http://medrxiv.org/lookup/doi/10.1101/2021.12.17.21267961.

[4] Yu, X. *et al.* Reduced sensitivity of SARS-CoV-2 Omicron variant to antibody neutralization elicited by booster vaccination. *Cell Discovery* **8**, 4 (2022).

URL https://www.nature.com/articles/s41421-022-00375-5. Publisher: Nature Publishing Group.

[5] Zhao, X. *et al.* Reduced sera neutralization to Omicron SARS-CoV-2 by both inactivated and protein subunit vaccines and the convalescents (2021). URL http://biorxiv.org/lookup/doi/10.1101/2021.12.16.472391.

[6] Zhao, X. *et al.* Effects of a Prolonged Booster Interval on Neutralization of Omicron Variant. *New England Journal of Medicine* **386**, 894–896 (2022). URL https://www.nejm.org/doi/full/10.1056/NEJMc2119426. Publisher: Massachusetts Medical Society _eprint: https://www.nejm.org/doi/pdf/10.1056/NEJMc2119426.

[7] Gruell, H. *et al.* Delineating antibody escape from Omicron sublineages (2022). URL http://biorxiv.org/lookup/doi/10.1101/2022.04.06.487257.

[8] Gruell, H. *et al.* SARS-CoV-2 Omicron sublineages exhibit distinct antibody escape patterns. *Cell Host & Microbe* **30**, 1231–1241.e6 (2022). URL https://www.cell.com/cell-host-microbe/abstract/S1931-3128(22)00318-3. Publisher: Elsevier.

[9] Kurhade, C. *et al.* Neutralization of Omicron sublineages and Deltacron SARS-CoV-2 by three doses of BNT162b2 vaccine or BA.1 infection. *Emerging Microbes & Infections* **11**, 1828–1832 (2022). URL https://doi.org/10.1080/22221751.2022.2099305. Publisher: Taylor & Francis _eprint: https://doi.org/10.1080/22221751.2022.2099305.

[10] Kurhade, C. *et al.* Neutralization of Omicron BA.1, BA.2, and BA.3 SARS-CoV-2 by 3 doses of BNT162b2 vaccine (2022). URL http://biorxiv.org/lookup/doi/10.1101/2022.03.24.485633.

[11] Dejnirattisai, W. *et al.* SARS-CoV-2 Omicron-B.1.1.529 leads to widespread escape from neutralizing antibody responses. *Cell* **185**, 467–484.e15 (2022). URL https://www.sciencedirect.com/science/article/pii/S0092867421015786.

[12] Stiasny, K. *et al.* Human primary Omicron BA.1 and BA.2 infections result in sub-lineage-specific neutralization (2022). URL https://www.researchsquare.com/article/rs-1536794/v1.

[13] Medits, I. *et al.* Different Neutralization Profiles After Primary SARS-CoV-2 Omicron BA.1 and BA.2 Infections. *Frontiers in Immunology* **13** (2022). URL https://www.frontiersin.org/journals/immunology/articles/10.3389/fimmu.2022.946318/full. Publisher: Frontiers.

[14] Chen, L.-L., Chu, A. W.-H., Zhang, R. R.-Q., Hung, I. F.-N. & To, K. K.-W. Serum neutralisation of the SARS-CoV-2 omicron sublineage BA.2. *The Lancet Microbe* **3**, e404 (2022). URL https://linkinghub.elsevier.com/retrieve/pii/S266652472200060X.

[15] Cao, Y. *et al.* Characterizations of enhanced infectivity and antibody evasion of Omicron BA.2.75 (2022). URL http://biorxiv.org/lookup/doi/10.1101/2022.07.18.500332.

[16] Cao, Y. *et al.* Characterization of the enhanced infectivity and antibody evasion of Omicron BA.2.75. *Cell Host & Microbe* **30**, 1527–1539.e5 (2022). URL https://www.cell.com/cell-host-microbe/abstract/S1931-3128(22)00511-X. Publisher: Elsevier.

[17] Kassambara, A. _rstatix: Pipe-Friendly Framework for Basic Statistical Tests_ (2023). URL https://CRAN.R-project.org/package=rstatix.
